# Markedly Divergent Tree Assemblage Responses to Tropical Forest Loss and Fragmentation across a Strong Seasonality Gradient

**DOI:** 10.1371/journal.pone.0136018

**Published:** 2015-08-26

**Authors:** Rodrigo L. L. Orihuela, Carlos A. Peres, Gabriel Mendes, João A. Jarenkow, Marcelo Tabarelli

**Affiliations:** 1 Departamento de Botânica, Universidade Federal do Rio Grande do Sul, Porto Alegre, Brazil; 2 School of Environmental Sciences, University of East Anglia, Norwich Research Park, Norwich, United Kingdom; 3 Departamento de Botânica, Universidade Federal de Pernambuco, Recife, Brazil; Technical University in Zvolen, SLOVAKIA

## Abstract

We examine the effects of forest fragmentation on the structure and composition of tree assemblages within three seasonal and aseasonal forest types of southern Brazil, including evergreen, Araucaria, and deciduous forests. We sampled three southernmost Atlantic Forest landscapes, including the largest continuous forest protected areas within each forest type. Tree assemblages in each forest type were sampled within 10 plots of 0.1 ha in both continuous forests and 10 adjacent forest fragments. All trees within each plot were assigned to trait categories describing their regeneration strategy, vertical stratification, seed-dispersal mode, seed size, and wood density. We detected differences among both forest types and landscape contexts in terms of overall tree species richness, and the density and species richness of different functional groups in terms of regeneration strategy, seed dispersal mode and woody density. Overall, evergreen forest fragments exhibited the largest deviations from continuous forest plots in assemblage structure. Evergreen, Araucaria and deciduous forests diverge in the functional composition of tree floras, particularly in relation to regeneration strategy and stress tolerance. By supporting a more diversified light-demanding and stress-tolerant flora with reduced richness and abundance of shade-tolerant, old-growth species, both deciduous and Araucaria forest tree assemblages are more intrinsically resilient to contemporary human-disturbances, including fragmentation-induced edge effects, in terms of species erosion and functional shifts. We suggest that these intrinsic differences in the direction and magnitude of responses to changes in landscape structure between forest types should guide a wide range of conservation strategies in restoring fragmented tropical forest landscapes worldwide.

## Introduction

The widespread conversion of old-growth tropical forests into small forest fragments [1], and mounting pressure to increase food production over the next decades is expected to further replace natural habitat with farmland in perhaps 1 billion hectares [2]. In fact, current and past rates of forest conversion clearly indicate that most unprotected old-growth tropical forests will be eventually phased out, leaving a complex mosaic of agricultural areas and forest fragments under varying successional stages [3], [4], challenging applied ecologists to examine organismal responses to human disturbance and the role played by anthropogenic landscapes as biodiversity repositories [5].

Overall, habitat loss and fragmentation can dramatically alter the composition and structure of tree assemblages across forest edges and edge-dominated small forest fragments [6], [7]. Changes in tree assemblage structure can be largely explained by either positive or negative abundance responses of functional groups sharing divergent life histories and morpho-ecological traits, such as seed-size, seed dispersal mode, and regeneration strategy [7]. Recent studies have shown that, compared to core mature forest areas, edge-dominated small fragments typically exhibit reduced abundances and species richness of emergent trees [8], [7], slow-growing, heavy-wooded trees [9], large-seeded vertebrate-dispersed plants [10], [11], shade-tolerant species sensitive to desiccation [12], and those exhibiting supra-annual flowering [13]. Conversely, fast-growing pioneer or successional species are predisposed to growth in light gaps, often proliferate at multiple spatial scales, and have been described as native ‘winners’ [14]. Ecological filtering imposed by physical edge effects, and dispersal limitation at multiple spatial scales may explain most shifts experienced by tree assemblages in human-modified landscapes [15], but plant species erosion may be further aggravated by fires, logging and climate change [13], [16].

Consequently, edge-dominated landscapes are likely to (1) retain only a small, non-random subset of the original flora [6], [17], (2) experience a proliferation of native pioneers and biotic homogenization [14], and (3) move further towards successional-systems [17], [18]. Despite these emergent patterns from studies across neotropical forests [6], [13], biological responses to habitat loss and fragmentation and the conservation value of human-modified landscapes remain obscure because tropical biotas differ in their physical environment, evolutionary history, and past and contemporary exposure to both natural and human-mediated disturbances [19], [20]. In other words, tropical floras differ in their natural abundance and diversity of disturbance-adapted or stress-tolerant species and, consequently, their inherent susceptibility to contemporary human disturbances. Yet fragmentation ecology research has been spatially concentrated in the neotropical region [20], particularly in evergreen forests (e.g. Projeto Dinâmica Biológica de Fragmentos Florestais (PDBFF) in Central Amazonia), and susceptibility to human disturbances have been traditionally addressed at species level (see syntheses in Bierregaard [21], [22]). Despite these potential limitations, comparisons across forest domains can elucidate functional response types to human disturbance as severe droughts induce forest die-offs [16], the land-use ‘agricultural bomb’ approaches [2], and relentless climate change proceeds apace.

The Atlantic Forest once covered ~150 million ha [23], straddling both tropical and subtropical regions of South America with a long history of highly heterogeneous baseline environmental conditions. Briefly, this unique biogeographic region consists of both seasonally-dry deciduous forests, away from the coast, and relatively aseasonal evergreen tropical rain forests subjected to more constant orographic precipitation near the coast. This once vast biome has been reduced to less than 12% of its original forest cover [23], and is currently one of the world’s most imperilled biodiversity hostpots [24]. The abundance of human-modified landscapes with a long history of human-disturbances associated with a relatively well-known regional biota in terms of both taxonomy and life-history attributes provide a key opportunity to address dynamic drivers of biodiversity persistence in modified landscapes for a myriad of taxa and ecological scenarios [13], [23], [25].

Here, we examine the effects of forest fragmentation on the structure and composition of tree assemblages across the three dominant forest types of the Atlantic Forest region of southern Brazil: evergreen, Araucaria, and deciduous forest. Assemblage attributes, such as species richness, species composition and functional groupings–within classes of regeneration strategy, forest stratification, seed dispersal mode and seed size–are compared between both habitat context (continuous forest vs. forest fragments) and forest types. We expected tangible differences between bioclimatic forest types in the abundance of functional groups affected by forest fragmentation, particularly due to baseline floristic differences in the abundance/diversity of stress-tolerant species as indicated in the description of these forest types [26–29]. Finally, we explore the theoretical and practical management implications of our findings.

## Methods

### 2.1. Study design

**Fig 1:**
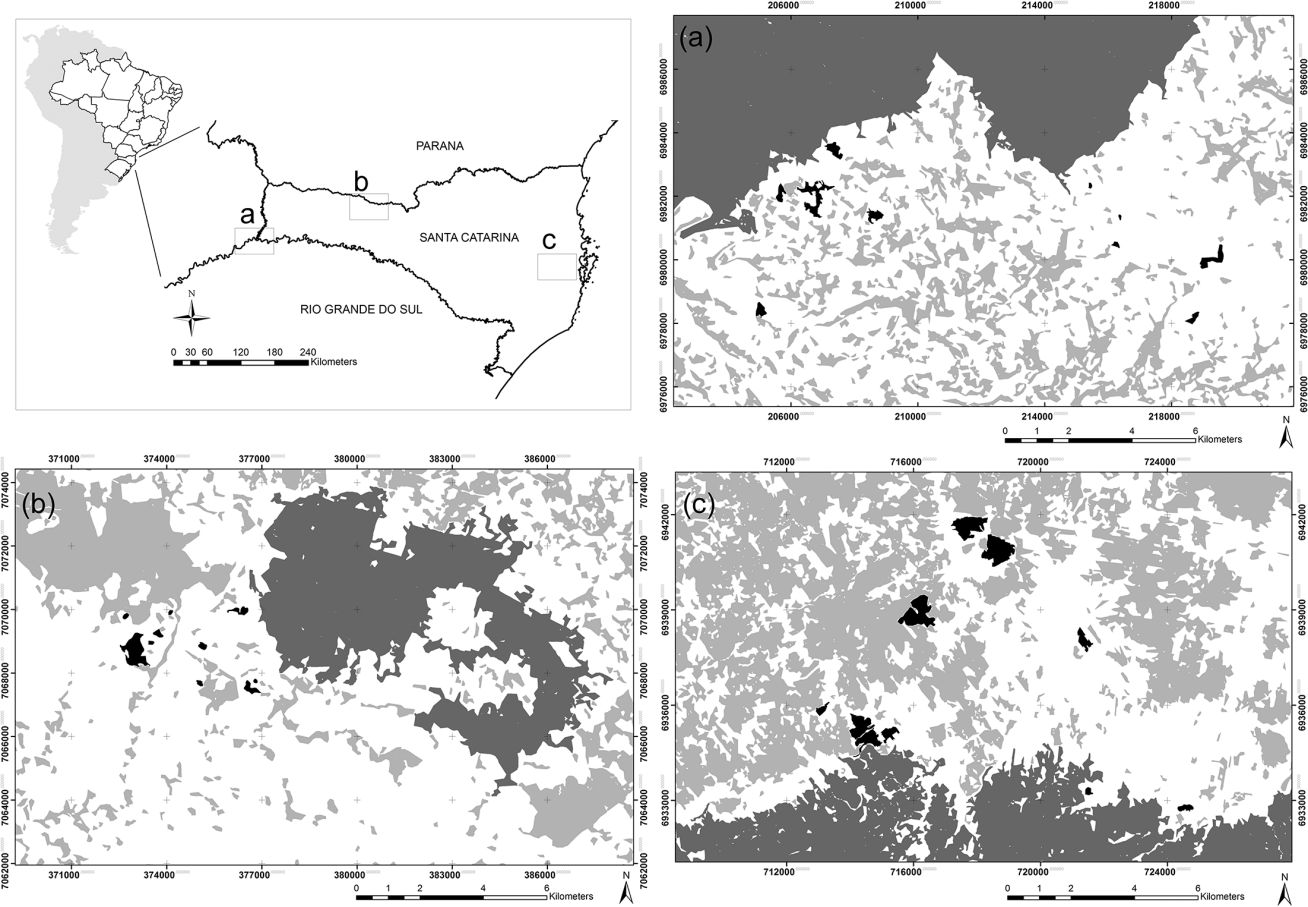
Landscapes observed in three different forest types in the Atlantic Forest domain of southern Brazil. Location of the three landscapes examined across three different forest types in the Atlantic Forest domain of southern Brazil. Large dark gray polygons represent the three protected areas used as control forests; small black polygons indicate the 10 forest fragments sampled in each landscape. Light gray areas refer to other forest fragments in the region. (a) Seasonal Deciduous Forest, including the Turvo State Park; (b) Araucaria Mixed Forests, including the Mata Preta Ecological Station; and (c) Evergreen or Moist/Ombrophilous Dense Forest, including the Serra do Tabuleiro State Park.

Our study encompassed three landscapes distributed across the three main southern Brazilian Atlantic Forest domains: evergreen, Araucaria, and deciduous forests (Fig 1). These forest types are referred to as Ombrophilous Dense Forest, Ombrophilous Mixed Forest, and Deciduous Forest, respectively, according to the Brazilian vegetation classification [30]. Deciduous trees account for fewer than 25% of all canopy species in evergreen forests but over half of the tree flora in deciduous forests [30]. In our study region, these three forest types span a wide gradient of seasonality in terms of both temperature and rainfall distribution, but mean total annual rainfall in all study areas is similar (1,500–2,000 mm yr^–1^).

In each forest type we selected one landscape containing one large patch of strictly protected old-growth forest used as a ‘pseudo-control’ in addition to 10 small forest patches (1.5 to 85 ha in size), which were located within 20 km from the perimeter of each pseudo-control forest area (so that forest fragment and control plots were similarly spaced in each study landscape). Landscape pre-selection was based on an extensive analysis of satellite imagery, field surveys and information provided by a digital map of Brazilian vegetation types [31]. Our study landscapes, which ranged from 176 to 486 km^2^ in size, spanned a geographic polygon of 82,800 km^2^. We next describe each landscape briefly.

#### Evergreen forest

The Serra do Tabuleiro State Park (STSP: 87,405 ha) served as a control since it is the largest protected area in this region and consists primarily of old-growth forest patches. The regional climate is humid mesothermal, with a hot summer and well distributed rainfall throughout the year (Cfa in Köppen-Geiser’s classification [32]). Elevations ranged from 220 to 490 m asl, mean rainfall is ~1500 mm/yr, and mean annual temperature is 20°C [33]. The non-forest matrix outside forest patches is dominated by pasture and cropland.

#### Araucaria forest

The Mata Preta Ecological Station (MPES: 6,563 ha) served as a control area as it is the last large remnant of Araucaria Forest in southern Brazil. Araucaria or candelabra tree are vernacular names of this iconic southern Brazilian conifer (*Araucaria angustifolia*, Araucariaceae), which forms the emergent stratum (up to 40 m) of this forest type, whereas canopy and understory plants typically consist of broadleaved Atlantic Forest species [34]. The regional climate is also moist subtropical with hot summers (Cfa in Köppen’s classification [32]), with a mean annual temperature of 18.7°C, a mean rainfall of 2,002 mm/yr, and regular frosts between June and August [35]. Cattle pastures and cropland dominate the landscape matrix which was once a vast continuous area of Araucaria forest.

#### Deciduous forest

The Turvo State Park (TSP: 17,491 ha), located in the north-western Rio Grande do Sul state, served as a control. All Atlantic Forest patches in this region are classified as deciduous forests [30]. TSP is the largest remaining contiguous old-growth tract of deciduous forest in southern Brazil [36]. The regional climate is subtropical sub-humid with relatively dry summers, with a mean rainfall of ~1,900 mm, and a marked hydrological deficit from late spring to early summer [37]. Frosts regularly occur between June and August and the mean annual temperature is 18–20°C [38]. Forest fragments sampled were embedded within the area surrounding the park, which had been largely converted into an intensively farmed landscape dominated by soybean and maize agriculture without any buffer zone protection.

Although these three landscapes have different histories of human disturbance, we only selected small forest fragments if they clearly lacked any evidence of human disturbance (e.g. wildfires, timber extraction, and bovine cattle intrusion). We randomly selected the first 10 fragments that met these requirements, and for which we obtained private authorization (from local landowners) to access their properties and deploy forest plots. Due to the landscape configuration available to us, our study design is limited to only a single forest landscape (i.e. no landscape replicates) per forest type. The Araucaria forest originally covered ~42.5% of the State of Santa Catarina, but this forest type has been reduced to only 1–4% of its original tracts since the expansion of modern agriculture [39]. The deciduous forest has experienced a similar land-use trajectory and the TSP is the only remaining regional-scale area of old-growth forest [36]. Despite these limitations, (1) the landscapes assessed here provide the last opportunity for a comparative analysis of the ecological biogeography of southern Brazilian forests, with the added bonus that (2) regional tree floras are well-documented taxonomically and ecologically (i.e. Flora Ilustrada Catarinense).

### 2.2. Tree assemblages

Tree assemblages were sampled across the three landscapes using 0.1 ha (10m x 100m) plots including 10 plots within each control area of core forest and one plot in each forest fragment, amounting to 60 plots (or 6 hectares) sampled. These relatively small plots have been widely adopted across the Atlantic Forest to detect fragmentation-related effects (Santos [7], [13]. Core forest plots were located at least 250 m from the nearest forest edge and with no detectable influence of forest borders as follows: (1) the 10 plots sampled at STSP were randomly placed at least 250 m from the reserve boundaries; (2) the 10 plots sampled at MPES were sited perpendicularly to, and at least 250 m from an unpaved road within the park; and (3) the 10 plots sampled at TSP were sited perpendicularly to, and at least 250 m from the two unpaved roads linking the two park entrances with the Uruguay River. Our definition of core forest areas follows Laurance et al. [40] who showed that different types of edge effects rarely penetrate 200 m from the nearest forest edge. One 0.1-ha plot was located at the geometric centre of each forest fragment as previously implemented elsewhere [7]. All plots were georeferenced using a handheld GPS. All field campaigns were conducted between January 2011 and July 2012. All trees ≥10 cm DBH were measured and identified in situ and voucher specimens of any ambiguous stems were collected, compared with vouchers deposited at the Federal University of Rio Grande do Sul (UFRGS) ICN Herbarium in Porto Alegre, and all vouchers with any remaining ambiguous identification were sent to family specialists.

### 2.3. Tree species attributes

All individuals were assigned to mutually exclusive categories of functional groups describing their seed size, vertical stratification, seed-dispersal mode, regeneration strategy, and wood density based on a comprehensive literature compilation, including books, papers, monographs of the regional flora and MSc and PhD dissertations [41], several volumes of the Flora Ilustrada Catarinense and Flora Fanerogâmica do Estado de São Paulo). These sources were supplemented by our own combined lifetime personal knowledge on the life-history traits of southern Brazilian tree species and online sources (e.g. Global Wood Density database [42]).

Functional groups of tree species were classified according to criteria adopted elsewhere in the Atlantic Forest [7], [43–45], as following: (a) Regeneration strategy–*Pioneer species* were defined as species requiring high light environments as viable regenerating sites such as forest edges and treefall gaps. This group included both large and long-lived pioneer species, in addition to short-lived pioneers (*sensu* Whitmore [46], and is equivalent to ‘successional species’ [6], [47]). *Shade-tolerant species* are those capable of regenerating in shaded environments, such as the shaded understory of old-growth forests. Juveniles of these species may survive in shade for several years; (b) Forest stratification – *Understory species* comprised small trees flowering and fruiting in the lowest forest layer (<11 m); *Canopy species* occurred in the forest subcanopy and canopy; and *Emergent species* occurred in the highest forest layer [48]. Seed-dispersal mode – *Vertebrate-dispersed species* are those bearing diaspores attached to a fleshy pulp, aril, or other features typically associated with vertebrate seed dispersal vectors; and *Abiotically-dispersed species* are those producing winged seeds, plumes, other wind-dispersal devices that slow the rate of seed fall, those dispersed entirely by free fall, or seeds propelled explosively from the fruit [49]; *Seed size of vertebrate-dispersed species* – seeds were grouped according to a logarithmic scale of seed size, considering its longest dimension: < 1mm; 1–5mm; 5–10mm; 10–20mm; ≥ 20mm, scored from 1 to 5, respectively. *Wood specific gravity* (WSG) or wood density–recorded according to Chave [50] and by consulting the species-specific regional-scale literature (e.g. Lorenzi [51]).

Tree assemblages within each plot were thus characterized in terms of the following attributes: (1) stem density; (2) tree species richness; and the percentage of (3) pioneer species; (4) pioneer stems; (5) emergent species; (6) canopy species; (7) understory species; (8) vertebrate-dispersed species; and (9) mean size of vertebrate-dispersed seeds.

### 2.4. Patch and landscape metrics

We used three spatial metrics as explanatory variables for changes in tree assemblage attributes: (i) forest patch area, the total forest area within each fragment or continuous forest; (ii) patch connectivity, expressed as the total amount of surrounding forest cover within a 1-km external matrix buffer from each patch; and (iii) distance to continuous forest, the straight-line distance from each fragment to the continuous forest within the large protected areas.

These metrics were generated using ArcGIS version 10.1, following a supervised classification of a set of three Landsat Thematic Mapper scenes (Landsat 5 TM, path 220/row 79 from 2010; path 222/row 78 and path 223/row 79 from 2011), which are freely available at Instituto Nacional de Pesquisas Espaciais (INPE, Brazil). We conducted image interpretation using the Maximum Likelihood method based on clearly distinguishable land cover classes. These classes ranged from five to nine, depending on landscape structure, and represented forested areas (dense continuous forest or forest fragments), forest on valleys (forest shaded by a topographic mask), shaded forest (forest shaded by clouds), agriculture (cropland and pastures), dense agriculture (dense cropland and tree plantations), water, sparse vegetation (soil not completely exposed), exposed soil, clouds, urban areas, and cloud shading. Although no reference points were taken in the field, our own experience throught the study area during field sampling allowed us to define training samples based on the visual identification of easily distinguishable land cover classes in each landscape for land cover validation. Our accuracy assessment based on observed land cover classes reached a high overall accuracy level of 94% in evergreen forest, 98% in Araucaria forest, and 97% in deciduous forest. Also, the Kappa coefficient (K) was higher than 93% in all three landscapes. Overall accuracy, Kappa coefficient, commission and omission errors and partition of training samples are summarized in S1 Table. Elevation for each plot was obtained from high-resolution digital topographic maps—the digital elevation surface available from the Shuttle Radar Topography Mission (SRTM). Soil types for each site and tree plot were obtained according to the Brazilian soil classification system [52].

### 2.5. Ethic Statements

Prior to the field campaigns of the project, we had obtained all appropriate agreements from state and federal authorities of the protected areas that we sampled (FATMA, number 053/2010 GERUC/DPEC; IBAMA number 18838–1, respectively). All field activities were conducted under the full rigor of Brazilian legislation (Law No. 5,197 of 3^rd^ January 1967 (see http://www.planalto.gov.br/ccivil_03/leis/L5197.htm). Explicit verbal permits to conduct fieldwork were also obtained from local landowners prior to accessing all private properties.

### 2.6. Data analysis

Differences in tree assemblage attributes were examined within each habitat context (continuous forests vs. fragments) and across the three forest types as follows: One-way ANOVAs followed by Tukey post-hoc comparisons for differences in stem density, species richness and functional species composition. Differences in stem density and species richness within seed size classes were examined using contingency tables (G-tests). Generalized linear models (GLMs) were used to detect the effects of explanatory variables on tree assemblage attributes considering all 60 plots. The following explanatory variables were considered: forest patch size, patch connectivity, distance to the nearest continuous forest, elevation, habitat context, and soil type. We excluded from final models any explanatory variables exhibiting high collinearity (r ≥ 0.7) and/or variables that were not significant in explaining any of the functional abundance responses (in the GLMs).

We performed non-metric multidimensional scaling (NMDS) ordinations of the 20 plots in each forest landscape using a Bray-Curtis (BC) dissimilarity matrix of species abundance [53]. We allowed the ordination to be rotated and centered, using the function *metaMDS* of the *vegan* package in R v. 2.15 [54] to perform the NMDS. The percentage of pioneer stems within each plot was regressed against the first two axes of the NMDS ordinations (which were subsequently re-run based on only one axis) to detect the influence of pioneer species on multivariate patterns of species composition and abundance within each landscape. To test the hypothesis that plots placed within either pseudo-control forests or forest fragments were taxonomically more similar to one another across the three landscapes/forest types, we constructed a BC similarity matrix of species composition across all plots, and calculated the average (± SE) pairwise BC values for three groups of comparisons: (1) between plots in forest fragments within any given landscape (*N* = 45); (2) between plots within the continuous forest site of each landscape (*N* = 45); and (3) between any continuous forest plots and any forest fragment plots (*N* = 100). We compared differences in BC dissimilarities between the groups using permutational multivariate analysis of variance (PERMANOVA), using PAST v. 2.11 [55], with 9,999 permutations and Bonferroni-corrected p-values. We performed an indicator species analyses (sensu [56]) based on the six sets of plots comparing the pseudo-control and forest fragment plots across each landscape. Indicator species are indicative of particular groups of sites characterizing each habitat type. Finally, we ran Mantel tests, through randomization tests (using the *rtest* function of the *ade4* package in R) to address potential spatial effects of plot location on their taxonomic similarity.

## Results

**Fig 2:**
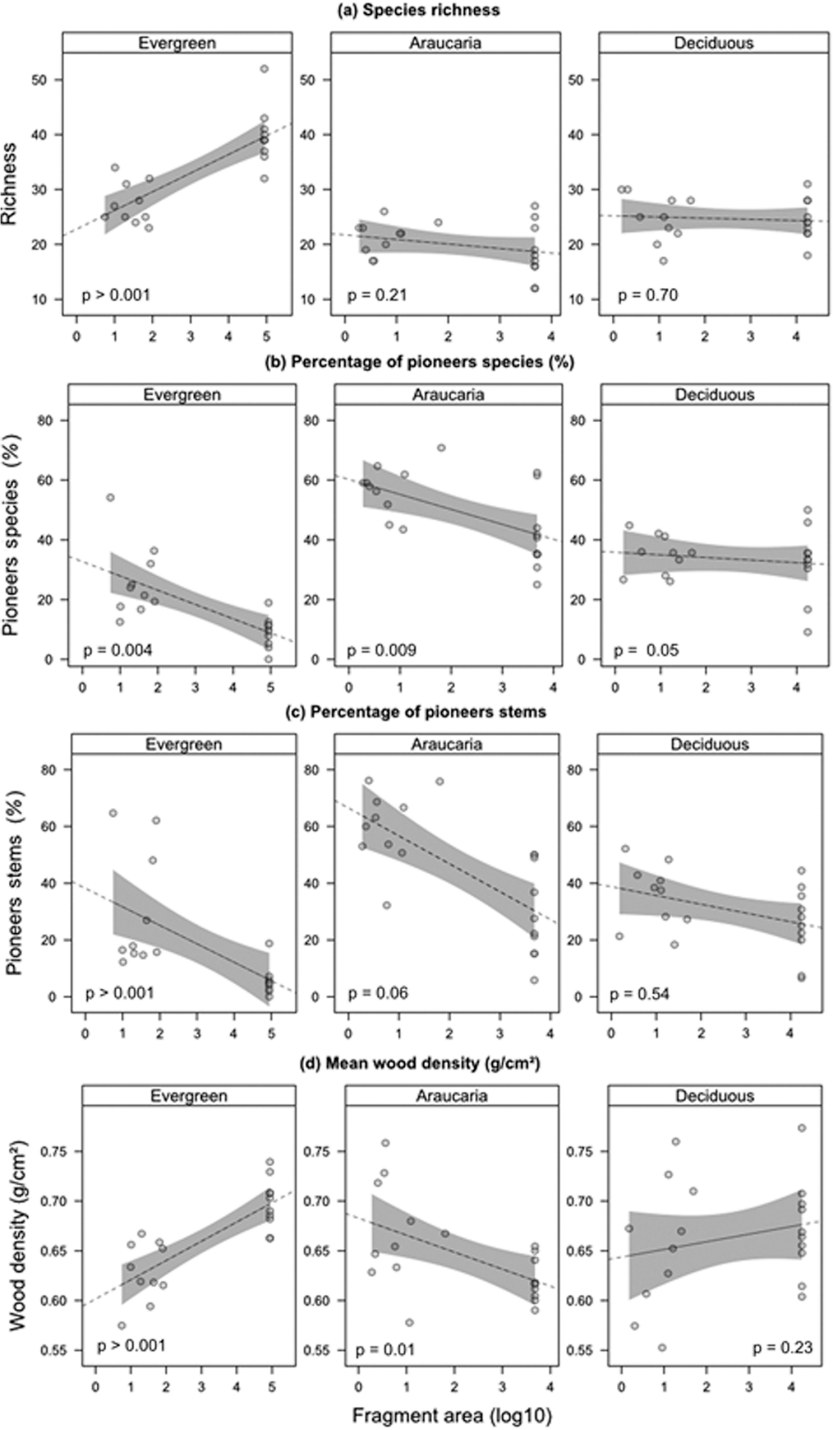
Relationships between forest patch area and tree species attributes. Relationships between log_10_ forest patch area (m²) and (a) tree species richness, (b) percentage of pioneer species, (c) percentage of pioneer stems, and (d) mean wood density (g/cm²) across three forest types of southern Brazil. Data points represent the 0.1-ha plots sampled in small forest fragments and core forest areas of continuous old-growth forest. P-values refer to linear regression models.

**Fig 3:**
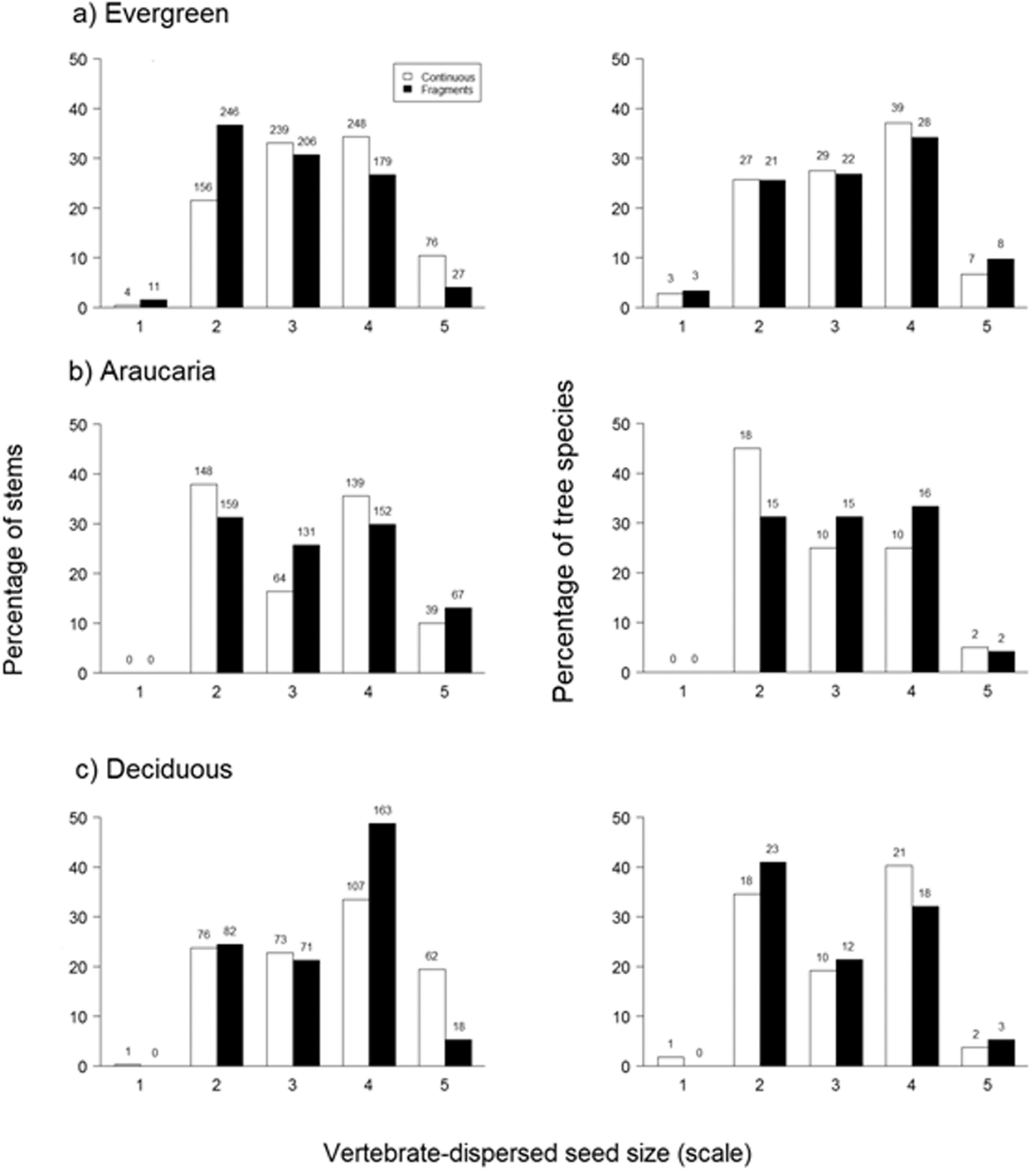
Percentage of tree stem abundance and species richness in relation to seed size classes across the three forest types of southern Brazil. Average percentage of stems and species in each category of vertebrate-dispersed seeds of different size classes in forest fragments (solid bars) and core areas of continuous old-growth forest (open bars) in three forest types. Numbers above each bar refer to the number of stems (left bars) and species (right bars). Seeds were grouped according to a logarithmic scale of seed size (from 1 to 5), considering their longest linear dimension: < 1 mm; 1–5 mm; 5–10 mm; 10–20 mm; ≥ 20mm.

A total of 3,972 trees belonging to 270 species and 72 families were recorded across all 60 plots. Evergreen forest plots exhibited the highest species richness (165), followed by deciduous forest (105) and Araucaria forest (87). Overall, control plots in continuous evergreen forest supported the most species-rich tree assemblages, but contained a lower proportion of pioneers, both in terms of species and individuals, compared to either Araucaria or deciduous forest plots (Table 1). There were also differences in the abundance and diversity of other functional groups between forest types (Table 1 and Fig 2). In particular, tree assemblages exhibited different responses to forest fragmentation across the three forest types (Table 1). There were marked differences between small fragments and continuous forest plots within all forest types in terms of stem density, tree species richness, the prevalence of pioneer species richness and abundance, and stem wood density. However, these shifts in assemblage structure were clearly more evident in evergreen forests, in which mean species richness in fragments was one-third lower and pioneer stems were six-fold more abundant (from 5.5% to nearly 30% of all stems) compared to continuous forest plots. Other attributes, such as the abundance of vertebrate-dispersed, large-seeded, and emergent trees, exhibited no detectable differences between fragments and continuous forests regardless of forest type, although evergreen forest fragments contained a higher proportion of stems bearing seeds >10 mm in length (Fig 3, G = 59.55, df = 4, p > 0.001). Finally, GLM models explained between 16% and 62% of the variation in tree assemblage attributes, which were affected by both forest type and forest patch area (Table 2). Again, stem density and species richness of taxa defined as pioneers exhibited marked responses to forest patch size, particularly in evergreen forest plots. Although overall tree species richness was higher in increasingly larger forest patches (Fig 2A), pioneer species increased in smaller patches regardless of forest type (Fig 2B and 2C). Finally, the amount of forest cover in the surrounding matrix had a significant negative effect on the stem density and diversity of pioneers (Table 2).

**Table 1:** Tree assemblage attributes (mean ± SD) in 0.1-ha plots located in forest fragments and core old-growth forest sites in the southern Brazilian Atlantic Forest biome. Seeds were grouped according to a logarithmic scale of seed size (from 1 to 5), considering their longest linear dimension: < 1 mm; 1–5 mm; 5–10 mm; 10–20 mm; ≥ 20mm.

Assemblage attributes	Evergreen		Araucaria		Deciduous		ANOVA	
	continuous	fragments	continuous	fragments	continuous	fragments		
	(n = 10)	(n = 10)	(n = 10)	(n = 10)	(n = 10)	(n = 10)	F	p
Stem density (per 0.1 ha)	93.3 ± 13.9 ^a^	74.5 ± 13.0 ^b^	65.8 ± 25.5 ^b,c^	65.0 ± 9.6 ^b,c^	46.3 ± 7.6 ^d^	52.3 ± 9.3 ^c,d^	20.36	**<0.0001**
Total species richness	39.8 ± 5.2 ^a^	27.4 ± 3.7 ^b^	18.5 ± 5.1 ^c^	21.3 ± 3.0 ^c,d^	24.5 ± 3.7 ^b,d^	24.8 ± 4.3 ^b,d^	30.22	**<0.0001**
Pioneer species (%)	9.1 ± 5.2 ^a^	23.3 ± 13.5 ^b^	41.2 ± 12.3 ^c^	57.0 ± 8.4 ^d^	32.2 ± 12.0 ^b,c^	35.0 ± 6.6 ^b,c^	25.46	**<0.0002**
Pioneer stems (%)	5.5 ± 5.1 ^a^	25.5 ± 20.5 ^b^	29.3 ± 16.2 ^b^	60.0 ± 13.3 ^c^	25.9 ± 12.4 ^b^	35.5 ± 11.3 ^b^	34.90	**<0.0001**
Emergent species (%)	5.5 ± 1.4	7.2 ± 3.7	8.4 ± 4.2	8.7 ± 3.0	9.0 ± 4.8	12.0 ± 6.6	4.09	**0.008**
Canopy species (%)	62.8 ± 8.0	64.7 ± 8.1	61.8 ± 7.1	61.4 ± 10.7	62.9 ± 3.7	62.1 ± 10.3	0.17	0.970
Understory species (%)	31.8 ± 7.3	28.1 ± 8.6	29.8 ± 7.4	29.9 ± 11.3	28.1 ± 4.4	25.8 ± 11.0	0.54	0.746
Vertebrate-dispersed species (%)	83.4 ± 4.5 ^a^	84.2 ± 3.3 ^a^	75.0 ± 6.8 ^a,b^	71.0 ± 9.0 ^b,c^	61.9 ± 9.5 ^c^	63.3 ± 13.3 ^c^	16.13	**<0.0001**
Vertebrate-dispersed seed size (scale)	3.3 ± 0.2 ^a,b^	2.9 ± 0.2 ^a^	3.2 ± 0.3 ^a,b^	3.3 ± 0.4 ^a,b^	3.5 ± 0.4 ^b^	3.4 ± 0.4 ^b^	3.49	0.008

Significant differences in post hoc comparisons (Tukey tests) between habitat types are indicated by different letters in the same row. Values in bold denote significant differences.

**Table 2:** Generalized linear model results explaining seven assemblage-wide tree species attributes across 60 plots sampled within three Atlantic forest types of southern Brazil. E = Evergreen forest; A = Araucaria forest; and D = Deciduous forest.

Assemblage attributes	Explanatory variables	Estimate (± SE)	z value	p-Value	R^2^ (%) whole model
Stem density (per 0.1 ha)	Forest type (E—A)	-0.310 (± 0.048)	-6.387	**< 0.0001**	0.529
	Forest type (E—D)	-0.600 (± 0.050)	-11.946	**< 0.0001**	
	Fragment area (log_10_ ha)	0.010 (± 0.010)	1.009	0.313	
	Surrounding forest cover	-0.348 (± 0.126)	-2.765	**0.006**	
					
Species richness	Forest type (E—A)	-0.594 (± 0.079)	-7.474	**< 0.0001**	0.609
	Forest type (E—D)	-0.370 (± 0.075)	-4.955	**< 0.0001**	
	Fragment area (log_10_ ha)	0.020 (± 0.016)	1.262	0.207	
	Surrounding forest cover	-0.464 (± 0.195)	-2.372	**0.017**	
					
Pioneer stems (%)	Forest type (E—A)	1.247 (± 0.102)	12.171	**< 0.0001**	0.620
	Forest type (E—D)	0.951 (± 0.114)	8.368	**< 0.0001**	
	Fragment area (log_10_ ha)	-0.156 (± 0.021)	-7.405	**< 0.0001**	
	Surrounding forest cover	1.397 (± 0.243)	5.755	**< 0.0001**	
					
Emergent stems (%)	Forest type (E—A)	0.468 (± 0.180)	2.603	**0.009**	0.178
	Forest type (E—D)	0.482 (± 0.187)	2.573	**0.010**	
	Fragment area (log_10_ ha)	-0.057 (± 0.036)	-1.58	0.114	
	Surrounding forest cover	-0.505 (± 0.454)	-1.114	0.265	
					
Vertebrate-dispersed species (%)	Forest type (E—A)	-0.158 (± 0.093)	-1.694	0.090	0.555
	Forest type (E—D)	-0.339 (± 0.090)	-3.784	**0.0001**	
	Fragment area (log_10_ ha)	0.001 (± 0.189)	0.04	0.968	
	Surrounding forest cover	-0.146 (± 0.231)	-0.631	0.528	
					
Vertebrate-dispersed seed size (scale)	Forest type (E—A)	0.132 (± 0.130)	1.015	0.314	0.167
	Forest type (E—D)	0.302 (± 0.128)	2.357	**0.022**	
	Fragment area (log_10_ ha)	0.045 (± 0.026)	1.727	0.090	
	Surrounding forest cover	-0.002 (± 0.325)	-0.006	0.995	
					
Mean wood density (g/cm^2^)	Forest type (E—A)	-0.009 (± 0.042)	-0.437	0.664	0.068
	Forest type (E—D)	0.008 (± 0.020)	0.393	0.696	
	Fragment area (log_10_ ha)	0.006 (± 0.004)	1.472	0.147	
	Surrounding forest cover	0.016 (± 0.050)	0.328	0.744	

**Fig 4:**
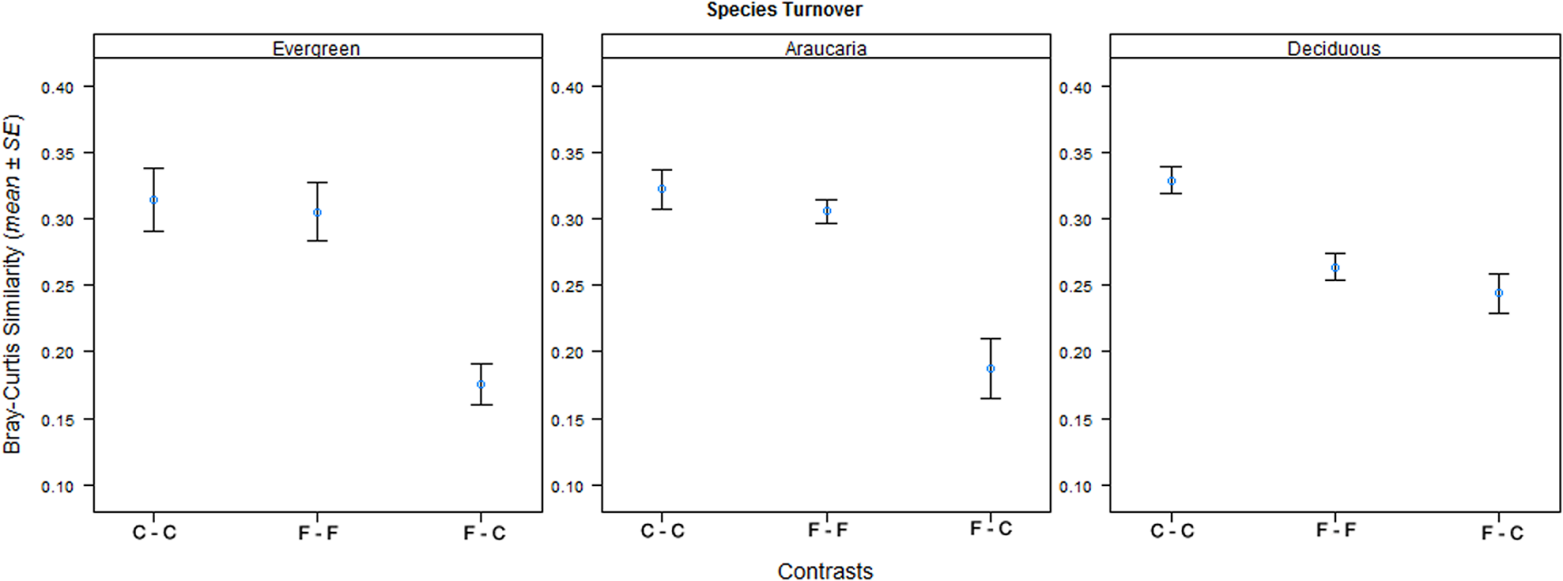
Bray-Curtis similarity between landscape contexts across the three forest types of southern Brazil. Bray-Curtis similarity values (mean ± SE) in species composition using abundance data both within and between landscape contexts across three forest types: between core forest plots (C-C); between forest fragment plots (F-F); and between forest fragment and core forest plots (F-C).

In terms of floristic composition considering both continuous forests and fragments, the three forest types exhibited marked baseline differences as the Myrtaceae, Lauraceae and Rubiaceae were more prevalent in evergreen forest (36% of all species), whereas Fabaceae, Myrtaceae, Rutaceae and Euphorbiaceae dominated deciduous forests (40% of all species), and Myrtaceae, Fabaceae and Lauraceae dominated Araucaria forests (29% of all species) (see S2 Table and S1 Dataset). Although the three forest types were only a few hundred kilometers apart (at most ~520 km), they shared only 4% of all species (12). Evergreen and deciduous forests shared 24 species (10%); Araucaria and deciduous forests shared 45 species (31%); and Araucaria and evergreen forests shared 30 species (14%). Within-habitat species similarity was relatively low and never exceeded 40%. Furthermore, pairwise comparisons between continuous forest and fragment plots exhibited very low levels of species similarity, ranging from 3.57 to 9.26% across the three forest types (Fig 4). Pairwise comparisons concerning species similarity between continuous forest and fragment plots exhibited significant differences in all three forest types (PERMANOVA, F = 8.918, p ≤ 0.05), with similarity scores between plots in continuous forest and fragments 30% lower than those within each forest habitat context (Fig 4). Mantel tests failed to uncover any large-scale spatial effects on the taxonomic similarity for 5 of the 6 groups of plots examined here (continuous and fragment forest plots across the three forest types). The proportion of species in each landscape restricted to continuous forest plots (i.e. missing in small fragments) ranged from a maximum of 40% in evergreen forest to only 18% in deciduous forest, with the latter exhibiting the highest levels of species similarity between continuous forest and fragment plots (F = 20.33, p < 0.0001).

**Fig 5:**
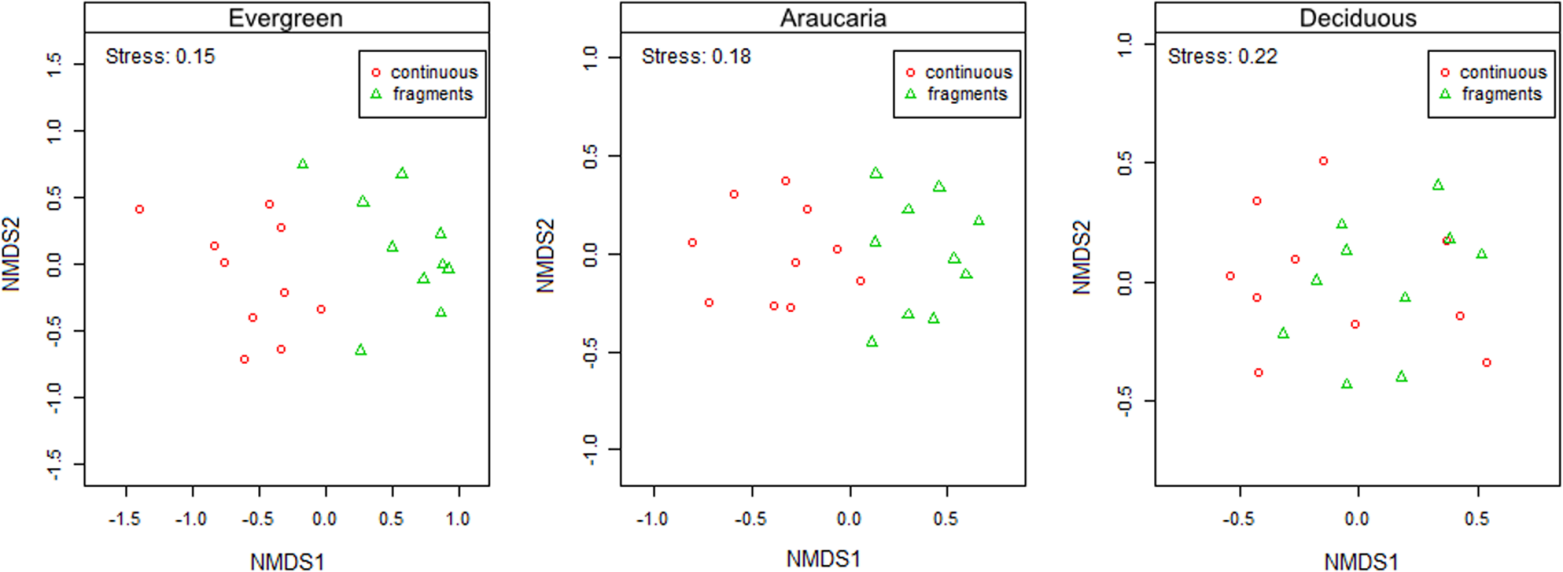
NMDS ordination of forest fragments and core continuous old-growth forest plots in southern Brazil. Non-metric multidimensional scaling (NMDS) ordination of sixty 0.1-ha plots located within forest fragments and core areas of continuous old-growth forest in each of the three forest types.

**Fig 6:**
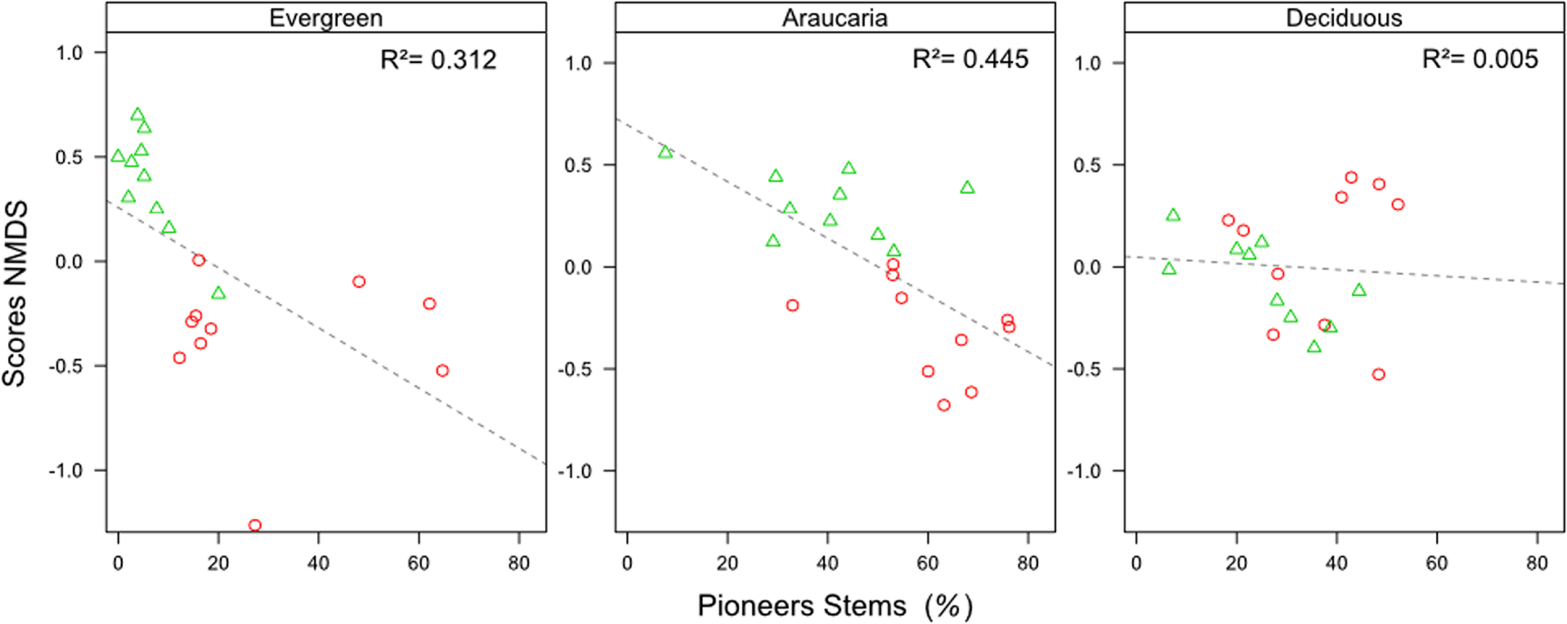
Relationship between NMDS ordination scores and the percentage of pioneer stems. Relationship between the NMDS scores (derived on only one axis) and the percentage of pioneer stems in 0.1-ha plots within forest fragments (red circles) and continuous forests (green triangles) in each of the three forest types. R^2^ values refer to linear regression models.

Differences in floristic responses to forest fragmentation across the three forest domains were confirmed by the NMDS ordination of tree assemblages. In short, two clusters of forest plots emerged separating the large core forest areas from small forest fragments. However, the spatial segregation of these clusters was much less evident in deciduous forest than in less seasonal forest types (Fig 5). These ordinations were supported by low stress levels ranging from 0.15 to 0.22. Linear regression models between the NMDS scores (derived on only one axis) describing community structure and the percentage of pioneer species occurring in each plot suggested that compositional changes across landscape contexts in evergreen and Araucaria forests are largely related to differences in pioneer abundance (R^2^ = 0.312 and 0.445, respectively; *p* values ≤ 0.01; Fig 6), but no such relationship was found in the seasonally-dry deciduous forest (R^2^ = 0.005; p = 0.770). Indicator species analyses within forest types underscored the occurrence of 56 species, most of which (24) in core evergreen forest. Small sets of indicator species emerged for evergreen forest fragments (8 species), Araucaria core forest (5), Araucaria forest fragments (9), and core deciduous forest (5) and fragments (5 species; S3 Table), with a strong tendency of shade-tolerant species being replaced by pioneers from core forest areas to small forest fragments across all three forest types. Note that only one pioneer species (a long-lived emergent) was identified as an indicator species across continuous forest plots, whereas 15 were detected in fragments. Conversely, 33 shade-tolerant species could be defined as indicators of core continuous forest plots, whereas only 7 species emerged as indicators of forest fragments.

## Discussion

Considering tree assemblages in tropical forests, only the most aseasonal sites studied here (evergreen forests) clearly support the notion that forest fragmentation (1) reduces tree species richness, (2) induces the proliferation of pioneer or successional species, (3) changes the stem abundance and species richness of different functional groups, and (4) drastically alters the taxonomic composition of tree assemblages, resulting in the emergence of a new set of indicator species [9], [40], [57], [58]. These taxonomic and functional shifts are usually correlated with both patch and landscape metrics [59], suggesting that community-wide shifts result from fragmentation-related processes, particularly responses to edge effects between core and edge-affected forest habitats, rather than baseline differences, such as soil types [7], [12], [60]. However, we documented marked differences between forest types in both the magnitude (number of attributes exhibiting shifts) and intensity (ecological deviation) of tree-assemblage responses to habitat fragmentation. As expected, these responses were consistent with a climatic gradient from relatively aseasonal to a markedly seasonal climate, and far more evident in evergreen forest than in either deciduous or Araucaria forest. A much larger proportion of species were shared between fragments and continuous forest in seasonal deciduous forest (Figs 4 and 5) compared to the two relatively aseasonal forest types. We particularly note the three-fold increase in the relative species richness of pioneers in evergreen forest fragments. The wider evergreen forest landscape that we sampled is relatively more forested than those in the other two forest types (Fig 1). However, we still find a markedly stronger detrimental response to forest loss and fragmentation in this aseasonal forest type. This therefore reinforces, rather than weakens, our main working hypothesis, as pioneer proliferation in edge-dominated forest habitat is typically suppressed by more forested landscapes (Table 2). Unfortunately, we were unable to replicate our sampling program across landscapes within each forest type due to the very low availability of remaining core continuous forest areas in southern Brazil that could serve as control forest sites. Abundance and compositional shifts in small forest remnants could therefore be potentially attributed to baseline differences associated, for instance, with soil type, landscape structure and historical land use [59]. However, these forest types are likely to be inherently different in their respective degrees of sensitivity to habitat fragmentation due to ecological and biogeographic differences in their baseline flora. As such while other neotropical studies have shown that tree assemblages in small evergreen forest fragments gradually lose old-growth tree species, become dominated by a small set of pioneer species, and are poorly represented in terms of tree functional groups typical of large tracts of primary forest, [6], [7], [40], [43], [61], this is the first study using a standardized sampling design to provide evidences that seasonal and aseasonal forests have clearly divergent responses to landscape change in terms of the functional and taxonomic shifts in the tree flora.

Tree species in southern Brazilian Araucaria and deciduous forests exhibit a higher proportion of light-demanding species as recorded in our study landscapes (51% and 49% of all species, respectively) compared to only 21% in evergreen forest. We differentiate two light-demanding regeneration guilds. The first typically consists of fast-growing, short- and long-lived pioneer species (sensu Whitmore [46]) or successional species with a natural population dynamics associated with treefall gaps in old-growth forest, but that can recruit in edge habitats [6]. Species within this group allocate higher investments to reproduction and population growth when inhabiting resource-unlimited habitats, compared to long-lived but shade-tolerant species that recruit in the forest understory. Such light-demanding forest species (including the genera *Albizia*, *Alchornea*, *Ceiba*, *and Cedrela*) take advantage of the highly illuminated understory underneath a more deciduous canopy in addition to the naturally discontinuous forest canopy in deciduous forest [62], and may benefit from elevated light levels and drier conditions in edge-affected habitats in forest fragments [58].

The second light-demanding tree strategy in Aracauria and deciduous forests includes species that can invade open-habitats such as the southern Brazilian grasslands (i.e. *campos sulinos*) and maintain viable populations in small forest patches and gallery forests, such as the natural “forest islands” of many semi-natural forest-grassland mosaics of southern Brazil [63], [64]. In these natural mosaics and ecotones, tree species are exposed to intense light exposure, extreme temperatures (including frosts), and water stress associated with droughts and shallow soils [27]. This light-demanding but stress-tolerant nucleating generalist flora exhibits high colonization ability [27], [65]. This guild is mainly represented by the candelabra tree (*Araucaria angustifolia*) and its affiliated species, some of which have biogeographic analogues in Austral-Antarctic and Andean floras [34], [66], and are related to other species shared with deciduous/semi-deciduous Atlantic forest patches [64]. These are represented by several species of *Sebastiana*, (Euphorbiaceae), *Solanum* (Solanaceae), *Parapiptadenia* (Leguminosae), and *Zanthoxylum* (Rutaceae), (see S2 Table; [65]).

Placing these life-history strategies into a conservation context, these light-demanding species operate as key drivers of forest expansion in southern Brazil [65]. These species may be defined as disturbance-adapted, by either lacking significant demographic responses or responding positively to habitat fragmentation as they have long been exposed to a wide evolutionary timescale amplitude in abiotic conditions that are comparable to those induced by edge creation in human-modified landscapes. Accordingly, the Araucaria and deciduous forest did not exhibit any increment in pioneer species richness or abundance in forest fragments despite modest shifts in taxonomic composition. In fact, it has long been proposed that plants with marked light-demanding strategies (1) are more tolerant to environmental stress induced by light exposure, water shortage and climatic extremes [67], [68], and (2) are expected to be poorly diversified and less abundant in evergreen forests, where shade-tolerance prevails due to predominantly humid and shaded conditions in old-growth forest [69]. Moreover, plant species in seasonally-dry forests should be inherently more tolerant to desiccation than those in evergreen forests [70], [71].

In synthesis, the evergreen, Araucaria and deciduous forests of the southern Atlantic Forest biome diverge in the ecological profile of their tree floras, particularly in terms of regeneration strategy and tolerance to microclimatic stress. By supporting a naturally more diversified light-demanding and stress-tolerant flora with reduced richness and abundance of shade-tolerant, old-growth species, both deciduous and Araucaria forests are expected to be more resilient to contemporary human disturbances, in terms of species erosion and functional shifts. In other words, these two forest types are intrinsically predisposed to be more tolerant to the emergence of human-modified landscapes due to an elevated number of disturbance-adapted species at multiple spatial scales, leading to some theoretical implications. Differences in sensitivity to habitat loss and fragmentation partly result from differences in the relative contribution of disturbance-adapted species to the baseline flora. In the case of tropical tree species, sensitivity to habitat fragmentation and associated disturbances involves life-history traits related to adult stature [40], sexual system and reproductive phenology, pollination and seed dispersal strategies [13], [47]. Additional life-history strategies may include physiological performance during environmental stress due to either resource limitation or climatic extremes. From an ecological perspective, seasonally-dry and transitional forests are expected to support a higher proportion of stress-tolerant species—that therefore confers them more resilience to forest fragmentation—than core old-growth evergreen forests and their extraordinary diversity of shade-tolerant woody plant species.

If corroborated by future studies elsewhere, our results have direct implications to the best conservation strategies whereby efforts employed in each forest domain should be guided by the evolutionary history of the tree flora. In relatively aseasonal forests, the preservation of large tracts of forest is crucial to retain most of the species diversity. This is consistent with several studies showing that small, edge-dominated forest patches are at best ill-suited to retain the bulk of the species diversity associated with moist, shaded environments of core, closed-canopy forests [15], [40], [43]. Conversely, setting-aside a large number of forest fragments (in addition to large forest reserves), of varying sizes can be considered a valid conservation strategy in seasonally-dry forests [72], [73]. Large continuous seasonal forest stands (>3,000 ha) are no longer available outside protected areas of southern Brazil, where >95% of the forest cover has been converted to agriculture and what remains is heavily degraded [74]. This is consistent with all seasonal tropical forests which currently retain only 2% of their original extent, are heavily fragmented [75], and are the most threatened forest type in any terrestrial biome [76], because of their high agricultural value due to high soil fertility and gentle slopes [77], [78]. Given this sorry state of affairs, we therefore end on a high note in extolling the conservation value of even small seasonally-dry forest fragments in retaining the biodiversity of the southern Atlantic forest biome. Finally, we encourage habitat managers worldwide to borrow from lessons in the evolutionary history to environmental stress of local biotas in informing large-scale conservation strategies. Ultimately, the evolution of local adaptations to environmental change, which are too often ignored, should be the primary canvas upon which contemporary conservation management guidelines should be designed or fine-tuned.

## Supporting Information

S1 Dataset Presence/absence data for all the 60 sampled plots, concerning all trees with DBH ≥ 10 cm, within three forest types of southern Brazil. (XLSX)

S1 Table Summary of accuracy assessment of land cover mapping in three forest types in southern Brazil: evergreen, Araucaria and deciduous forests. (XLSX)

S2 Table Abundance of all sampled tree species occurring in forest fragments and core old-growth continuous forest at three. A total of 10 forest plots of 0.1 ha each were sampled for each landscape context in each forest type. F = fragments; C = continuous forest plots.
(DOCX)

S3 Table Indicator species analysis of tree assemblages recorded in old-growth continuous forest and fragment plots in three forest types (n = 60 plots) in southern Brazil. (DOCX)
