# Supplementary material for: Markedly Divergent Tree Assemblage Responses to Tropical Forest Loss and Fragmentation across a Strong Seasonality Gradient
Source: PLoS One. 2015 Aug 26;10(8):e0136018. doi: 10.1371/journal.pone.0136018 (PMC4550385; doi:10.1371/journal.pone.0136018)
Supplement: S2 Table — A total of 10 forest plots of 0.1 ha each were sampled for each landscape context in each forest type. F = fragments; C = continuous forest plots. (DOCX) [file pone.0136018.s003.docx]

Table S2. Abundance of all sampled tree species occurring in forest fragments and core old-growth continuous forest at three forest types in southern Brazil. A total of 10 forest plots of 0.1 ha each were sampled for each landscape context in each forest type. F = fragments; C = continuous forest plots.

|  | | Evergreen | | | | Araucaria | | | Deciduous | | | |
| --- | --- | --- | --- | --- | --- | --- | --- | --- | --- | --- | --- | --- |
| Species | | F | | C | | F C | | C | F | | C | |
| *Achatocarpus praecox* Griseb. | 0 | | 0 | | 0 | | 0 | 6 | | 0 | |  |
| *Actinostemon concolor*(Spreng.) Müll.Arg. | 0 | | 3 | | 0 | | 0 | 0 | | 10 | |  |
| *Aegiphila brachiata* Vell. | 0 | | 0 | | 0 | | 0 | 3 | | 0 | |  |
| *Agonandra excelsa* Griseb. | 0 | | 0 | | 0 | | 0 | 1 | | 0 | |  |
| *Aiouea saligna*Meisn. | 3 | | 2 | | 0 | | 0 | 0 | | 0 | |  |
| *Albizia edwallii*(Hoehne) Barneby & J.W. Grimes | 1 | | 1 | | 0 | | 0 | 2 | | 2 | |  |
| *Albizia niopoides* (Spruce ex Benth.) Burkart | 0 | | 0 | | 0 | | 0 | 0 | | 1 | |  |
| *Alchornea glandulosa*Poepp. | 9 | | 0 | | 0 | | 0 | 0 | | 0 | |  |
| *Alchornea triplinervia*(Spreng.) Müll. Arg. | 8 | | 5 | | 2 | | 0 | 0 | | 2 | |  |
| *Allophylus edulis*(A.St.-Hil., Cambess. & A. Juss.) Radlk. | 0 | | 0 | | 4 | | 2 | 6 | | 9 | |  |
| *Allophylus puberulus*(Cambess.) Radlk. | 0 | | 0 | | 1 | | 0 | 0 | | 0 | |  |
| *Aloysia virgata* (Ruiz & Pav.) Juss. | 0 | | 0 | | 0 | | 0 | 11 | | 2 | |  |
| *Alsophila setosa*Kaulf. | 3 | | 38 | | 0 | | 24 | 8 | | 0 | |  |
| *Amaioua intermedia*Mart. | 11 | | 8 | | 0 | | 0 | 0 | | 0 | |  |
| *Andira fraxinifolia*Benth. | 4 | | 1 | | 0 | | 0 | 0 | | 0 | |  |
| *Aniba firmula*(Nees & C. Mart.) Mez | 0 | | 5 | | 0 | | 0 | 0 | | 0 | |  |
| *Annona cacans*Warm. | 1 | | 0 | | 0 | | 0 | 0 | | 0 | |  |
| *Annona neosalicifolia* H. Rainer | 0 | | 0 | | 10 | | 0 | 9 | | 6 | |  |
| *Annona neosericea*H. Rainer | 13 | | 11 | | 0 | | 0 | 0 | | 0 | |  |
| *Annona rugulosa* (Schltdl.) H. Rainer | 2 | | 0 | | 3 | | 3 | 2 | | 0 | |  |
| *Aparisthmium cordatum*(A. Juss.) Baill. | 0 | | 3 | | 0 | | 0 | 0 | | 0 | |  |
| *Apuleia leiocarpa* (Vogel) J.F. Macbr. | 0 | | 0 | | 0 | | 0 | 8 | | 7 | |  |
| *Aralia warmingiana* (Marchal) J. Wen | 0 | | 0 | | 0 | | 0 | 1 | | 4 | |  |
| *Araucaria angustifolia*(Bertol.) Kuntze | 0 | | 0 | | 62 | | 37 | 0 | | 0 | |  |
| *Aspidosperma australe* Müll. Arg. | 1 | | 53 | | 0 | | 1 | 4 | | 0 | |  |
| *Attalea dubia*(Mart.) Burret | 2 | | 0 | | 0 | | 0 | 0 | | 0 | |  |
| *Balfourodendron riedelianum* (Engl.) Engl. | 0 | | 0 | | 0 | | 0 | 9 | | 11 | |  |
| *Banara tomentosa* Clos | 0 | | 0 | | 2 | | 9 | 2 | | 2 | |  |
| *Bathysa australis*(A. St.-Hil.) Hook. f. ex K. Schum. | 14 | | 14 | | 0 | | 0 | 0 | | 0 | |  |
| *Bauhinia forficata* Link | 0 | | 0 | | 0 | | 0 | 4 | | 0 | |  |
| *Brosimum lactescens* (S. Moore) C.C.Berg | 2 | | 1 | | 0 | | 0 | 0 | | 0 | |  |
| *Butia eriospatha*(Mart. ex Drude) Becc. | 0 | | 0 | | 1 | | 0 | 0 | | 0 | |  |
| *Byrsonima ligustrifolia*A. Juss. | 1 | | 19 | | 0 | | 0 | 0 | | 0 | |  |
| *Cabralea canjerana* (Vell.) Mart. | 12 | | 8 | | 0 | | 0 | 26 | | 9 | |  |
| *Calliandra foliolosa* Benth. | 0 | | 0 | | 0 | | 0 | 3 | | 2 | |  |
| *Callisthene kuhlmannii* H.F. Martins | 0 | | 1 | | 0 | | 0 | 0 | | 0 | |  |
| *Calyptranthes concinna*DC. | 0 | | 0 | | 19 | | 0 | 0 | | 0 | |  |
| *Calyptranthes grandifolia*O. Berg | 0 | | 17 | | 0 | | 0 | 0 | | 0 | |  |
| *Calyptranthes lucida*Mart. ex DC. | 1 | | 16 | | 0 | | 0 | 0 | | 0 | |  |
| *Calyptranthes strigipes*O. Berg | 1 | | 12 | | 0 | | 0 | 0 | | 0 | |  |
| *Calyptranthes tricona* D. Legrand | 0 | | 0 | | 0 | | 0 | 0 | | 26 | |  |
| *Campomanesia guazumifolia* (Cambess.) O.Berg | 0 | | 0 | | 5 | | 0 | 0 | | 3 | |  |
| *Campomanesia xanthocarpa* O. Berg | 0 | | 0 | | 55 | | 3 | 8 | | 3 | |  |
| *Campomanesia guaviroba*(DC.) Kiaersk. | 1 | | 0 | | 0 | | 0 | 0 | | 0 | |  |
| *Cinnamodendron dinisii*Schwanke | 0 | | 0 | | 3 | | 6 | 0 | | 0 | |  |
| *Casearia decandra* Jacq. | 0 | | 0 | | 22 | | 15 | 5 | | 1 | |  |
| *Casearia obliqua*Spreng. | 1 | | 5 | | 33 | | 20 | 0 | | 0 | |  |
| *Casearia sylvestris* Sw. | 15 | | 2 | | 3 | | 1 | 8 | | 5 | |  |
| *Cecropia glaziovii*Snethl. | 5 | | 1 | | 0 | | 0 | 0 | | 0 | |  |
| *Cedrela fissilis* Vell. | 6 | | 6 | | 8 | | 1 | 21 | | 8 | |  |
| *Ceiba speciosa* (A. St.-Hil.) Ravenna | 0 | | 0 | | 0 | | 0 | 0 | | 4 | |  |
| *Chionanthus filiformis*(Vell.) P.S. Green | 0 | | 3 | | 0 | | 0 | 0 | | 0 | |  |
| *Chrysophyllum gonocarpum* (Mart. & Eichler ex Miq.) Engl. | 0 | | 0 | | 0 | | 0 | 15 | | 23 | |  |
| *Chrysophyllum inornatum*Mart. | 0 | | 3 | | 0 | | 0 | 0 | | 0 | |  |
| *Chrysophyllum marginatum* (Hook. & Arn.) Radlk. | 0 | | 0 | | 0 | | 0 | 5 | | 24 | |  |
| *Chrysophyllum viride* Mart. & Eichler | 0 | | 2 | | 0 | | 0 | 0 | | 0 | |  |
| *Cinnamomum amoenum*(Nees & Mart.) Kosterm. | 0 | | 0 | | 4 | | 0 | 0 | | 0 | |  |
| *Cinnamomum glaziovii*(Mez) Kosterm. | 2 | | 1 | | 0 | | 0 | 0 | | 0 | |  |
| *Citharexylum myrianthum* Cham. | 1 | | 0 | | 0 | | 0 | 0 | | 0 | |  |
| *Citronella paniculata* (Mart.) R.A. Howard | 0 | | 0 | | 0 | | 0 | 0 | | 2 | |  |
| *Clethra scabra*Pers. | 5 | | 1 | | 11 | | 1 | 0 | | 0 | |  |
| *Clusia criuva*Cambess. | 13 | | 9 | | 0 | | 0 | 0 | | 0 | |  |
| *Coccoloba warmingii*Meisn. | 0 | | 2 | | 0 | | 0 | 0 | | 0 | |  |
| *Colubrina glandulosa*Perkins | 1 | | 1 | | 0 | | 0 | 0 | | 0 | |  |
| *Copaifera trapezifolia*Hayne | 0 | | 2 | | 0 | | 0 | 0 | | 0 | |  |
| *Cordia americana* (L.) Gottschling & J.S.Mill. | 0 | | 0 | | 2 | | 0 | 1 | | 0 | |  |
| *Cordia ecalyculata* Vell. | 0 | | 0 | | 0 | | 0 | 7 | | 2 | |  |
| *Cordia trichotoma* (Vell.) Arráb. ex Steud. | 0 | | 0 | | 0 | | 1 | 12 | | 3 | |  |
| *Cordiera concolor*(Cham.) Kuntze | 0 | | 5 | | 0 | | 3 | 0 | | 0 | |  |
| *Coussapoa microcarpa* (Schott) Rizzini | 1 | | 0 | | 0 | | 0 | 0 | | 0 | |  |
| *Coussarea contracta*(Walp.) Müll.Arg. | 1 | | 4 | | 11 | | 47 | 0 | | 0 | |  |
| *Coutarea hexandra*(Jacq.) K. Schum. | 0 | | 0 | | 0 | | 2 | 0 | | 0 | |  |
| *Cryptocarya aschersoniana*Mez | 10 | | 8 | | 0 | | 0 | 0 | | 0 | |  |
| *Cupania vernalis* Cambess. | 8 | | 1 | | 40 | | 64 | 27 | | 0 | |  |
| *Cyathea corcovadensis*(Raddi) Domin | 2 | | 8 | | 0 | | 0 | 0 | | 0 | |  |
| *Cyathea delgadii*Sternb. | 0 | | 10 | | 0 | | 0 | 0 | | 0 | |  |
| *Cyathea phalerata* Mart. | 0 | | 5 | | 0 | | 0 | 0 | | 0 | |  |
| *Cybistax antisyphilitica* (Mart.) Mart. | 1 | | 1 | | 0 | | 0 | 0 | | 0 | |  |
| *Dalbergia frutescens* (Vell.) Britton | 2 | | 0 | | 2 | | 0 | 8 | | 7 | |  |
| *Dasyphyllum spinescens*(Less.) Cabrera | 0 | | 0 | | 2 | | 0 | 0 | | 0 | |  |
| *Diatenopteryx sorbifolia* Radlk. | 0 | | 0 | | 2 | | 4 | 4 | | 8 | |  |
| *Dicksonia sellowiana*Hook. | 0 | | 0 | | 2 | | 172 | 0 | | 0 | |  |
| *Diospyros inconstans* Jacq. | 0 | | 0 | | 0 | | 0 | 0 | | 2 | |  |
| *Drimys brasiliensis*Miers | 0 | | 0 | | 0 | | 2 | 0 | | 0 | |  |
| *Duguetia lanceolata*A. St.-Hil. | 0 | | 12 | | 0 | | 0 | 0 | | 0 | |  |
| *Enterolobium contortisiliquum* (Vell.) Morong | 0 | | 0 | | 0 | | 0 | 1 | | 0 | |  |
| *Erythrina falcata* Benth. | 0 | | 0 | | 0 | | 0 | 1 | | 1 | |  |
| *Erythroxylum deciduum* A.St.-Hil. | 0 | | 0 | | 1 | | 0 | 1 | | 0 | |  |
| *Esenbeckia grandiflora* Mart. | 0 | | 1 | | 0 | | 0 | 0 | | 0 | |  |
| *Eugenia beaurepairiana*(Kiaersk.) D. Legrand | 0 | | 5 | | 0 | | 0 | 0 | | 0 | |  |
| *Eugenia burkartiana* (D. Legrand) D. Legrand | 1 | | 0 | | 0 | | 0 | 0 | | 0 | |  |
| *Eugenia cereja* D. Legrand | 1 | | 0 | | 0 | | 0 | 0 | | 0 | |  |
| *Eugenia excelsa*O. Berg | 0 | | 1 | | 0 | | 0 | 0 | | 0 | |  |
| *Eugenia handroana* D. Legrand | 0 | | 1 | | 0 | | 0 | 0 | | 0 | |  |
| *Eugenia hiemalis* Cambess. | 0 | | 0 | | 0 | | 0 | 0 | | 1 | |  |
| *Eugenia involucrata* DC. | 0 | | 0 | | 6 | | 0 | 0 | | 1 | |  |
| *Eugenia multicostata*D. Legrand | 0 | | 1 | | 0 | | 0 | 0 | | 0 | |  |
| *Eugenia nutans* O. Berg | 0 | | 1 | | 0 | | 0 | 0 | | 0 | |  |
| *Eugenia pyriformis* Cambess. | 0 | | 0 | | 12 | | 0 | 1 | | 1 | |  |
| *Eugenia ramboi* D.Legrand | 0 | | 0 | | 0 | | 2 | 1 | | 0 | |  |
| *Eugenia subterminalis* DC. | 0 | | 0 | | 0 | | 0 | 1 | | 0 | |  |
| *Eugenia uniflora* L. | 0 | | 0 | | 24 | | 0 | 5 | | 1 | |  |
| *Euterpe edulis*Mart. | 66 | | 18 | | 0 | | 0 | 0 | | 0 | |  |
| *Faramea montevidensis*(Cham. & Schltdl.) DC. | 3 | | 3 | | 0 | | 0 | 0 | | 0 | |  |
| *Ficus adhatodifolia*Schott ex Spreng. | 5 | | 1 | | 0 | | 0 | 0 | | 0 | |  |
| *Ficus arpazusa* Casar. | 1 | | 0 | | 0 | | 0 | 0 | | 0 | |  |
| *Ficus citrifolia* Mill. | 0 | | 0 | | 0 | | 0 | 0 | | 1 | |  |
| *Ficus luschnathiana* (Miq.) Miq. | 2 | | 0 | | 0 | | 0 | 3 | | 1 | |  |
| *Ficus organensis* Miq. | 0 | | 2 | | 0 | | 0 | 0 | | 0 | |  |
| *Garcinia gardneriana* (Planch. & Triana) Zappi | 1 | | 7 | | 0 | | 0 | 0 | | 0 | |  |
| *Gleditsia amorphoides* (Griseb.) Taub. | 0 | | 0 | | 0 | | 0 | 1 | | 0 | |  |
| *Guapira opposita*(Vell.) Reitz | 11 | | 40 | | 0 | | 0 | 0 | | 0 | |  |
| *Guarea macrophylla* Vahl | 5 | | 0 | | 0 | | 0 | 0 | | 1 | |  |
| *Guatteria australis* A. St.-Hil. | 6 | | 8 | | 0 | | 0 | 0 | | 0 | |  |
| *Hedyosmum brasiliense*Miq. | 0 | | 2 | | 0 | | 0 | 0 | | 0 | |  |
| *Heisteria silvianii*Schwacke | 2 | | 7 | | 0 | | 0 | 0 | | 0 | |  |
| *Helietta apiculata* Benth. | 0 | | 0 | | 0 | | 0 | 12 | | 6 | |  |
| *Heliocarpus popayanensis* Kunth | 0 | | 0 | | 0 | | 0 | 0 | | 1 | |  |
| *Hennecartia omphalandra* J.Poiss. | 0 | | 0 | | 0 | | 0 | 1 | | 0 | |  |
| *Hirtella hebeclada*Moric. ex DC. | 3 | | 51 | | 0 | | 0 | 0 | | 0 | |  |
| *Holocalyx balansae* Micheli | 0 | | 0 | | 0 | | 0 | 11 | | 15 | |  |
| *Hovenia dulcis* Thunb. | 0 | | 0 | | 0 | | 1 | 0 | | 0 | |  |
| *Hyeronima alchorneoides*Allemão | 74 | | 13 | | 0 | | 0 | 0 | | 0 | |  |
| *Ilex brevicuspis*Reissek | 3 | | 0 | | 2 | | 1 | 0 | | 0 | |  |
| *Ilex dumosa*Reissek | 0 | | 1 | | 0 | | 0 | 0 | | 0 | |  |
| *Ilex microdonta*Reissek | 0 | | 0 | | 2 | | 4 | 0 | | 0 | |  |
| *Ilex paraguariensis*A. St.-Hil. | 0 | | 5 | | 4 | | 23 | 0 | | 0 | |  |
| *Ilex taubertiana* Loes. | 0 | | 1 | | 0 | | 0 | 0 | | 0 | |  |
| *Ilex theezans*Mart. ex Reissek | 11 | | 3 | | 3 | | 1 | 0 | | 0 | |  |
| *Inga marginata* Willd. | 2 | | 1 | | 0 | | 0 | 2 | | 4 | |  |
| *Inga vera* Willd. | 0 | | 0 | | 0 | | 2 | 0 | | 2 | |  |
| *Inga virescens*Benth. | 0 | | 0 | | 1 | | 0 | 0 | | 0 | |  |
| *Jacaranda micrantha*Cham. | 2 | | 0 | | 0 | | 1 | 0 | | 0 | |  |
| *Jacaranda puberula*Cham. | 6 | | 1 | | 6 | | 2 | 0 | | 0 | |  |
| *Jacaratia spinosa* (Aubl.) A.DC. | 0 | | 0 | | 0 | | 0 | 1 | | 4 | |  |
| *Lamanonia ternata*Vell. | 0 | | 3 | | 27 | | 12 | 0 | | 0 | |  |
| *Laplacea fruticosa*(Schrad.) Kobuski | 1 | | 0 | | 0 | | 0 | 0 | | 0 | |  |
| *Lithraea brasiliensis*Marchand | 0 | | 0 | | 21 | | 1 | 0 | | 0 | |  |
| *Lonchocarpus campestris* Mart. ex Benth. | 0 | | 0 | | 0 | | 0 | 4 | | 7 | |  |
| *Luehea divaricata* Mart. & Zucc. | 0 | | 0 | | 3 | | 1 | 12 | | 16 | |  |
| *Machaerium hirtum* (Vell.) Stellfeld | 0 | | 0 | | 0 | | 0 | 0 | | 1 | |  |
| *Machaerium nyctitans*(Vell.) Benth. | 0 | | 1 | | 0 | | 0 | 0 | | 0 | |  |
| *Machaerium paraguariense* Hassl. | 0 | | 0 | | 1 | | 1 | 2 | | 3 | |  |
| *Machaerium stipitatum* (DC.) Vogel | 0 | | 0 | | 1 | | 2 | 15 | | 11 | |  |
| *Maclura tinctoria* (L.) D.Don ex Steud. | 4 | | 0 | | 0 | | 0 | 1 | | 5 | |  |
| *Magnolia ovata*(A. St.-Hil.) Spreng. | 2 | | 6 | | 0 | | 0 | 0 | | 0 | |  |
| *Manihot grahamii* Hook. | 0 | | 0 | | 0 | | 0 | 2 | | 0 | |  |
| *Marlierea excoriata*Mart. | 2 | | 2 | | 0 | | 0 | 0 | | 0 | |  |
| *Marlierea sylvatica*(Gardner) Kiaersk. | 2 | | 4 | | 0 | | 0 | 0 | | 0 | |  |
| *Matayba elaeagnoides* Radlk. | 0 | | 0 | | 14 | | 19 | 3 | | 0 | |  |
| *Matayba intermedia*Radlk. | 29 | | 16 | | 0 | | 0 | 0 | | 0 | |  |
| *Maytenus muelleri*Schwacke | 0 | | 0 | | 0 | | 1 | 0 | | 0 | |  |
| *Maytenus robusta* Reissek | 1 | | 9 | | 0 | | 0 | 0 | | 0 | |  |
| *Meliosma sellowii*Urb. | 0 | | 7 | | 0 | | 0 | 0 | | 0 | |  |
| *Miconia cabussu*Hoehne | 44 | | 12 | | 0 | | 0 | 0 | | 0 | |  |
| *Miconia cinnamomifolia*(DC.) Naudin | 41 | | 4 | | 0 | | 0 | 0 | | 0 | |  |
| *Miconia cubatanensis*Hoehne | 0 | | 1 | | 0 | | 0 | 0 | | 0 | |  |
| *Miconia pusilliflora* (DC.) Naudin | 0 | | 0 | | 0 | | 0 | 1 | | 0 | |  |
| *Mimosa scabrella*Benth. | 0 | | 0 | | 3 | | 0 | 0 | | 0 | |  |
| *Mollinedia triflora* (Spreng.) Tul. | 1 | | 1 | | 0 | | 0 | 0 | | 0 | |  |
| *Myrcia brasiliensis*Kiaersk. | 7 | | 4 | | 0 | | 0 | 0 | | 0 | |  |
| *Myrcia dichrophylla*D. Legrand | 0 | | 10 | | 0 | | 0 | 0 | | 0 | |  |
| *Myrcia glabra*(O.Berg) D. Legrand | 2 | | 0 | | 0 | | 0 | 0 | | 0 | |  |
| *Myrcia guianensis* (Aubl.) DC. | 0 | | 3 | | 2 | | 0 | 0 | | 0 | |  |
| *Myrcia oblongata* DC. | 0 | | 0 | | 51 | | 0 | 0 | | 0 | |  |
| *Myrcia pubipetala* Miq. | 12 | | 15 | | 0 | | 0 | 0 | | 0 | |  |
| *Myrcia pulchra* (O. Berg) Kiaersk. | 0 | | 1 | | 3 | | 0 | 0 | | 0 | |  |
| *Myrcia racemosa*(O. Berg) Kiaersk. | 0 | | 2 | | 0 | | 0 | 0 | | 0 | |  |
| *Myrcia richardiana*(O. Berg) Kiaersk. | 0 | | 3 | | 0 | | 0 | 0 | | 0 | |  |
| *Myrcia selloi*(Spreng.) N. Silveira | 0 | | 0 | | 1 | | 0 | 0 | | 0 | |  |
| *Myrcia spectabilis*DC. | 0 | | 2 | | 0 | | 0 | 0 | | 0 | |  |
| *Myrcia splendens*(Sw.) DC. | 5 | | 0 | | 0 | | 0 | 0 | | 0 | |  |
| *Myrcia tijucensis* Kiaersk. | 1 | | 8 | | 0 | | 0 | 0 | | 0 | |  |
| *Myrciaria floribunda*(West ex Willd.) O. Berg | 3 | | 0 | | 3 | | 0 | 0 | | 0 | |  |
| *Myrocarpus frondosus*  Allemão | 0 | | 0 | | 0 | | 0 | 3 | | 2 | |  |
| *Myrsine coriacea*(Sw.) R. Br. ex Roem. & Schult | 3 | | 0 | | 1 | | 9 | 0 | | 0 | |  |
| *Myrsine guianensis* (Aubl.) Kuntze | 0 | | 0 | | 0 | | 2 | 1 | | 2 | |  |
| *Myrsine umbellata* Mart. | 0 | | 2 | | 0 | | 0 | 0 | | 0 | |  |
| *Nectandra oppositifolia*Nees & Mart. | 0 | | 0 | | 4 | | 1 | 19 | | 0 | |  |
| *Nectandra megapotamica* (Spreng.) Mez | 0 | | 0 | | 4 | | 26 | 20 | | 4 | |  |
| *Nectandra oppositifolia*Nees & Mart. | 22 | | 10 | | 0 | | 0 | 0 | | 0 | |  |
| *Neomitranthes glomerata*(D. Legrand) D. Legrand | 0 | | 1 | | 0 | | 0 | 0 | | 0 | |  |
| *Ocotea aciphylla*(Nees) Mez | 1 | | 13 | | 0 | | 0 | 0 | | 0 | |  |
| *Ocotea catharinensis Mez* | 0 | | 7 | | 0 | | 0 | 0 | | 0 | |  |
| *Ocotea corymbosa* (Meisn.) Mez | 0 | | 2 | | 0 | | 0 | 0 | | 0 | |  |
| *Ocotea diospyrifolia* (Meisn.) Mez | 0 | | 0 | | 6 | | 45 | 5 | | 5 | |  |
| *Ocotea elegans*Mez | 0 | | 2 | | 0 | | 0 | 0 | | 0 | |  |
| *Ocotea indecora*(Schott) Mez | 0 | | 4 | | 0 | | 0 | 0 | | 0 | |  |
| *Ocotea lancifolia* (Schott) Mez | 0 | | 6 | | 0 | | 0 | 0 | | 0 | |  |
| *Ocotea mandioccana*A. Quinet | 1 | | 2 | | 0 | | 0 | 0 | | 0 | |  |
| *Ocotea nectandrifolia*Mez | 0 | | 7 | | 0 | | 0 | 0 | | 0 | |  |
| *Ocotea odorifera* Rohwer | 0 | | 3 | | 0 | | 0 | 0 | | 0 | |  |
| *Ocotea porosa* (Nees & Mart.) Barroso | 0 | | 1 | | 0 | | 0 | 0 | | 0 | |  |
| *Ocotea puberula* (Rich.) Nees | 8 | | 0 | | 5 | | 3 | 8 | | 0 | |  |
| *Ocotea pulchella*Mart. | 0 | | 1 | | 14 | | 0 | 0 | | 0 | |  |
| *Ocotea pulchra*Vattimo-Gil | 0 | | 2 | | 0 | | 0 | 0 | | 0 | |  |
| *Ocotea silvestris*Vattimo | 0 | | 5 | | 0 | | 0 | 0 | | 0 | |  |
| *Ormosia arborea*(Vell.) Harms | 2 | | 3 | | 0 | | 0 | 0 | | 0 | |  |
| *Ouratea sellowii* Engl. | 0 | | 2 | | 0 | | 0 | 0 | | 0 | |  |
| *Parapiptadenia rigida* (Benth.) Brenan | 0 | | 0 | | 3 | | 1 | 5 | | 4 | |  |
| *Pausandra morisiana*(Casar.) Radlk. | 0 | | 2 | | 0 | | 0 | 0 | | 0 | |  |
| *Peltophorum dubium* (Spreng.) Taub. | 0 | | 0 | | 0 | | 0 | 1 | | 0 | |  |
| *Pera glabrata*(Schott) Poepp. ex Baill. | 60 | | 37 | | 0 | | 0 | 0 | | 0 | |  |
| *Phytolacca dioica* L. | 0 | | 0 | | 0 | | 0 | 3 | | 0 | |  |
| *Picramnia parvifolia* Engl. | 0 | | 0 | | 0 | | 0 | 0 | | 1 | |  |
| *Picrasma crenata* (Vell.) Engl. | 0 | | 0 | | 0 | | 1 | 2 | | 4 | |  |
| *Pilocarpus pennatifolius* Lem. | 0 | | 0 | | 0 | | 0 | 6 | | 4 | |  |
| *Pimenta pseudocaryophyllus* (Gomes) Landrum | 0 | | 2 | | 0 | | 0 | 0 | | 0 | |  |
| *Piptadenia gonoacantha*(Mart.) J.F.Macbr. | 1 | | 0 | | 0 | | 0 | 0 | | 0 | |  |
| *Piptocarpha angustifolia* Dusén ex Malme | 0 | | 2 | | 2 | | 14 | 0 | | 0 | |  |
| *Piptocarpha sellowii* (Sch. Bip.) Baker | 4 | | 0 | | 9 | | 5 | 2 | | 0 | |  |
| *Pisonia zapallo* Griseb. | 1 | | 4 | | 0 | | 0 | 3 | | 5 | |  |
| *Plinia cordifolia* (D. Legrand) Sobral | 0 | | 2 | | 0 | | 0 | 0 | | 0 | |  |
| *Plinia edulis*(Vell.) Sobral | 2 | | 0 | | 0 | | 0 | 0 | | 0 | |  |
| *Plinia rivularis* (Cambess.) Rotman | 0 | | 0 | | 0 | | 0 | 2 | | 2 | |  |
| *Podocarpus sellowii* Klotzsch ex Endl. | 0 | | 1 | | 0 | | 0 | 0 | | 0 | |  |
| *Posoqueria latifolia*(Rudge) Roem. & Schult. | 5 | | 13 | | 0 | | 0 | 0 | | 0 | |  |
| *Pouteria venosa*(Mart.) Baehni | 1 | | 0 | | 0 | | 0 | 0 | | 0 | |  |
| *Protium kleinii*Cuatrec. | 0 | | 28 | | 0 | | 0 | 0 | | 0 | |  |
| *Prunus myrtifolia* (L.) Urb. | 0 | | 0 | | 18 | | 10 | 18 | | 2 | |  |
| *Psychotria carthagenensis*Jacq. | 0 | | 15 | | 0 | | 0 | 0 | | 0 | |  |
| *Psychotria suterella*Müll. Arg. | 0 | | 1 | | 0 | | 0 | 0 | | 0 | |  |
| *Psychotria vellosiana*Benth. | 25 | | 8 | | 0 | | 0 | 0 | | 0 | |  |
| *Qualea cryptantha*(Spreng.) Warm. | 0 | | 1 | | 0 | | 0 | 0 | | 0 | |  |
| *Qualea minor*(Mart.) Spreng. | 0 | | 1 | | 0 | | 0 | 0 | | 0 | |  |
| *Quiina glazovii*  Engl. | 1 | | 1 | | 0 | | 0 | 0 | | 0 | |  |
| *Randia ferox* (Cham. & Schltdl.) DC. | 0 | | 0 | | 0 | | 0 | 1 | | 1 | |  |
| *Roupala montana*Aubl. | 1 | | 7 | | 2 | | 0 | 0 | | 0 | |  |
| *Rudgea jasminoides*(Cham.) Müll. Arg. | 0 | | 3 | | 0 | | 0 | 0 | | 0 | |  |
| *Rudgea recurva*Müll. Arg | 0 | | 4 | | 0 | | 0 | 0 | | 0 | |  |
| *Ruprechtia laxiflora* Meisn. | 0 | | 0 | | 1 | | 1 | 2 | | 2 | |  |
| *Sapium glandulosum*(L.) Morong | 0 | | 0 | | 14 | | 0 | 0 | | 0 | |  |
| *Schefflera angustissima* (Marchal) Frodin | 0 | | 8 | | 0 | | 0 | 0 | | 0 | |  |
| *Schefflera calva* (Cham.) Frodin & Fiaschi | 1 | | 0 | | 0 | | 0 | 4 | | 2 | |  |
| *Schinus terebinthifolius Raddi* | 0 | | 0 | | 1 | | 0 | 0 | | 0 | |  |
| *Sebastiania brasiliensis Spreng.* | 0 | | 0 | | 0 | | 0 | 14 | | 17 | |  |
| *Sebastiania commersoniana* (Baill.) L.B.Sm. & Downs | 0 | | 0 | | 9 | | 0 | 5 | | 6 | |  |
| *Seguieria aculeata*Jacq. | 0 | | 0 | | 0 | | 1 | 0 | | 0 | |  |
| *Seguieria langsdorffii*Moq. | 1 | | 0 | | 0 | | 0 | 0 | | 0 | |  |
| *Sloanea guianensis*(Aubl.) Benth. | 32 | | 34 | | 0 | | 0 | 0 | | 0 | |  |
| *Sloanea monosperma* Vell. | 0 | | 1 | | 0 | | 4 | 0 | | 0 | |  |
| *Solanum pseudoquina* A.St.-Hil. | 0 | | 0 | | 0 | | 0 | 2 | | 0 | |  |
| *Solanum sanctae-catharinae* Dunal | 0 | | 0 | | 1 | | 1 | 1 | | 0 | |  |
| *Sorocea bonplandii*(Baill.) W.C. Burger, Lanjouw & Boer | 0 | | 6 | | 0 | | 0 | 8 | | 15 | |  |
| *Strychnos brasiliensis*(Spreng.) Mart. | 0 | | 0 | | 2 | | 1 | 0 | | 0 | |  |
| *Styrax acuminatus*Pohl | 1 | | 0 | | 0 | | 0 | 0 | | 0 | |  |
| *Styrax leprosus* Hook. & Arn. | 0 | | 0 | | 15 | | 7 | 3 | | 5 | |  |
| *Syagrus romanzoffiana* (Cham.) Glassman | 2 | | 0 | | 3 | | 2 | 15 | | 60 | |  |
| *Symplocos tenuifolia*Brand | 0 | | 0 | | 0 | | 7 | 0 | | 0 | |  |
| *Symplocos tetrandra* Mart. | 0 | | 0 | | 0 | | 1 | 2 | | 0 | |  |
| *Symplocos trachycarpa*Brand | 2 | | 0 | | 0 | | 0 | 0 | | 0 | |  |
| *Syzygium jambos*(L.) Alston | 1 | | 0 | | 0 | | 0 | 0 | | 0 | |  |
| *Tabernaemontana catharinensis* A.DC. | 3 | | 0 | | 0 | | 0 | 0 | | 3 | |  |
| *Tapirira guianensis*Aubl. | 4 | | 0 | | 0 | | 0 | 0 | | 0 | |  |
| *Tetrorchidium rubrivenium* Poepp. | 4 | | 0 | | 0 | | 0 | 3 | | 3 | |  |
| *Trema micrantha* (L.) Blume | 0 | | 0 | | 0 | | 1 | 2 | | 2 | |  |
| *Trichilia catigua* A.Juss. | 0 | | 0 | | 0 | | 0 | 3 | | 7 | |  |
| *Trichilia claussenii* C.DC. | 0 | | 0 | | 0 | | 0 | 17 | | 20 | |  |
| *Trichilia lepidota*Mart. | 3 | | 2 | | 0 | | 0 | 0 | | 0 | |  |
| *Urera baccifera* (L.) Gaudich. ex Wedd. | 0 | | 0 | | 0 | | 0 | 23 | | 4 | |  |
| *Vantanea compacta* (Schnizl.) Cuatrec. | 0 | | 29 | | 0 | | 0 | 0 | | 0 | |  |
| *Vasconcellea quercifolia* A.St.-Hil. | 0 | | 0 | | 0 | | 0 | 2 | | 0 | |  |
| *Vernonanthura discolor* (Spreng.) H.Rob. | 0 | | 0 | | 26 | | 8 | 0 | | 0 | |  |
| *Virola bicuhyba*(Schott ex Spreng.) Warb. | 6 | | 8 | | 0 | | 0 | 0 | | 0 | |  |
| *Vitex megapotamica* (Spreng.) Moldenke | 0 | | 0 | | 2 | | 4 | 1 | | 1 | |  |
| *Xylopia brasiliensis* Spreng. | 8 | | 19 | | 0 | | 0 | 0 | | 0 | |  |
| *Xylosma pseudosalzmannii* Sleumer | 0 | | 0 | | 0 | | 0 | 2 | | 2 | |  |
| *Zanthoxylum fagara* (L.) Sarg. | 0 | | 0 | | 0 | | 0 | 1 | | 1 | |  |
| *Zanthoxylum petiolare* A.St.-Hil. & Tul. | 0 | | 0 | | 0 | | 0 | 0 | | 1 | |  |
| *Zanthoxylum rhoifolium* Lam. | 1 | | 0 | | 1 | | 0 | 0 | | 1 | |  |
