# Supplementary material for: Markedly Divergent Tree Assemblage Responses to Tropical Forest Loss and Fragmentation across a Strong Seasonality Gradient
Source: PLoS One. 2015 Aug 26;10(8):e0136018. doi: 10.1371/journal.pone.0136018 (PMC4550385; doi:10.1371/journal.pone.0136018)
Supplement: S3 Table — (DOCX) [file pone.0136018.s004.docx]

Table S3. Indicator species analysis of tree assemblages recorded in old-growth continuous forest and fragment plots in three forest types (n=60 plots) in southern Brazil.

| Indicator species | Family | Regeneration strategy | Indicator value (IV) | *p* |
| --- | --- | --- | --- | --- |
| Evergreen - continuous |  |  |  |  |
| *Hirtella hebeclada* | Chrysobalanaceae | s-Tolerant | 0.972 | 0.001 |
| *Aspidosperma australe* | Apocynaceae | s-Tolerant | 0.899 | 0.001 |
| *Protium kleinii* | Burseraceae | s-Tolerant | 0.894 | 0.001 |
| *Calyptranthes lucida* | Myrtaceae | s-Tolerant | 0.868 | 0.001 |
| *Schefflera angustissima* | Araliaceae | s-Tolerant | 0.837 | 0.001 |
| *Vantanea compacta* | Humiriaceae | s-Tolerant | 0.837 | 0.001 |
| *Byrsonima ligustrifolia* | Malpighiaceae | s-Tolerant | 0.815 | 0.001 |
| *Calyptranthes grandifolia* | Myrtaceae | s-Tolerant | 0.775 | 0.001 |
| *Duguetia lanceolata* | Annonaceae | s-Tolerant | 0.775 | 0.001 |
| *Calyptranthes strigipes* | Myrtaceae | s-Tolerant | 0.744 | 0.001 |
| *Maytenus robusta* | Celastraceae | s-Tolerant | 0.735 | 0.001 |
| *Myrcia tijucensis* | Myrtaceae | s-Tolerant | 0.73 | 0.002 |
| *Eugenia beaurepairiana* | Myrtaceae | s-Tolerant | 0.707 | 0.001 |
| *Meliosma sellowii* | Sabiaceae | s-Tolerant | 0.707 | 0.002 |
| *Myrcia dichrophylla* | Myrtaceae | s-Tolerant | 0.707 | 0.001 |
| *Heisteria silvianii* | Olacaceae | s-Tolerant | 0.683 | 0.001 |
| *Ocotea aciphylla* | Lauraceae | s-Tolerant | 0.681 | 0.003 |
| *Garcinia gardneriana* | Clusiaceae | s-Tolerant | 0.661 | 0.002 |
| *Aniba firmula* | Lauraceae | s-Tolerant | 0.632 | 0.003 |
| *Ocotea catharinensis* | Lauraceae | s-Tolerant | 0.632 | 0.004 |
| *Ocotea indecora* | Lauraceae | s-Tolerant | 0.632 | 0.004 |
| *Ocotea nectandrifolia* | Lauraceae | s-Tolerant | 0.632 | 0.006 |
| *Ocotea silvestris* | Lauraceae | s-Tolerant | 0.548 | 0.024 |
| *Rudgea recurva* | Rubiaceae | s-Tolerant | 0.548 | 0.028 |
| Evergreen - fragments |  |  |  |  |
| *Miconia cinnamomifolia* | Melastomataceae | Pioneer | 0.799 | 0.001 |
| *Miconia cabussu* | Melastomataceae | s-Tolerant | 0.793 | 0.001 |
| *Psychotria vellosiana* | Rubiaceae | s-Tolerant | 0.778 | 0.001 |
| *Ilex theezans* | Aquifoliaceae | Pioneer | 0.654 | 0.001 |
| *Alchornea glandulosa* | Euphorbiaceae | Pioneer | 0.632 | 0.002 |
| *Myrcia splendens* | Myrtaceae | Pioneer | 0.548 | 0.017 |
| *Cecropia glaziovii* | Urticaceae | Pioneer | 0.500 | 0.049 |
| *Guarea macrophylla* | Meliaceae | s-Tolerant | 0.500 | 0.048 |
| Araucaria - continuous |  |  |  |  |
| *Dicksonia sellowiana* | Dicksoniaceae | s-Tolerant | 0.832 | 0.001 |
| *Ocotea diospyrifolia* | Lauraceae | s-Tolerant | 0.815 | 0.001 |
| *Coussarea contracta* | Rubiaceae | s-Tolerant | 0.773 | 0.002 |
| *Ilex paraguariensis* | Aquifoliaceae | s-Tolerant | 0.758 | 0.001 |
| *Symplocos tenuifolia* | Symplocaceae | s-Tolerant | 0.548 | 0.026 |
| Araucaria - fragments |  |  |  |  |
| *Myrcia oblongata* | Myrtaceae | Pioneer | 0.894 | 0.001 |
| *Campomanesia xanthocarpa* | Myrtaceae | s-Tolerant | 0.847 | 0.001 |
| *Sapium glandulosum* | Euphorbiaceae | Pioneer | 0.837 | 0.001 |
| *Eugenia pyriformis* | Myrtaceae | s-Tolerant | 0.717 | 0.001 |
| *Lithraea brasiliensis* | Anacardiaceae | Pioneer | 0.691 | 0.001 |
| *Calyptranthes concinna* | Myrtaceae | Pioneer | 0.632 | 0.003 |
| *Ocotea pulchella* | Lauraceae | Pioneer | 0.611 | 0.006 |
| *Eugenia uniflora* | Myrtaceae | Pioneer | 0.566 | 0.017 |
| *Albizia niopoides* | Fabaceae | Pioneer | 0.548 | 0.016 |
| Seasonal - continuous |  |  |  |  |
| *Chrysophyllum marginatum* | Sapotaceae | s-Tolerant | 0.863 | 0.001 |
| *Gymnanthes concolor* | Euphorbiaceae | s-Tolerant | 0.62 | 0.003 |
| *Trichilia catigua* | Meliaceae | s-Tolerant | 0.592 | 0.003 |
| *Calyptranthes tricona* | Myrtaceae | s-Tolerant | 0.548 | 0.024 |
| *Ceiba speciosa* | Malvaceae | Pioneer | 0.548 | 0.019 |
| Seasonal - fragments |  |  |  |  |
| *Cordia trichotoma* | Boraginaceae | s-Tolerant | 0.671 | 0.001 |
| *Urera baccifera* | Urticaceae | Pioneer | 0.653 | 0.002 |
| *Bauhinia forficata* | Fabaceae | Pioneer | 0.632 | 0.001 |
| *Cordia ecalyculata* | Boraginaceae | s-Tolerant | 0.624 | 0.007 |
| *Achatocarpus praecox* | Achatocarpaceae | Pioneer | 0.548 | 0.022 |
